# Wearable Sensors and Artificial Intelligence for Sleep Apnea Detection: A Systematic Review

**DOI:** 10.1007/s10916-025-02199-8

**Published:** 2025-05-19

**Authors:** Ainhoa Osa-Sanchez, Javier Ramos-Martinez-de-Soria, Amaia Mendez-Zorrilla, Ibon Oleagordia Ruiz, Begonya Garcia-Zapirain

**Affiliations:** eVIDA Research Group, University of Deusto, Bilbao, 48007 Spain

**Keywords:** Sleep apnea disease, Wearable devices, Artificial intelligence, Biomedical data

## Abstract

Sleep apnea, a prevalent disorder affecting millions of people worldwide, has attracted increasing attention in recent years due to its significant impact on public health and quality of life. The integration of wearable devices and artificial intelligence technologies has revolutionized the treatment and diagnosis of sleep apnea. Leveraging the portability and sensors of wearable devices, coupled with AI algorithms, has enabled real-time monitoring and accurate analysis of sleep patterns, facilitating early detection and personalized interventions for people suffering from sleep apnea. This article presents a systematic review of the current state of the art in identifying the latest artificial intelligence techniques, wearable devices, data types, and preprocessing methods employed in the diagnosis of sleep apnea. Four databases were used and the results before screening report 249 studies published between 2020 and 2024. After screening, 28 studies met the inclusion criteria. This review reveals a trend in recent years where methodologies involving patches, clocks and rings have been increasingly integrated with convolutional neural networks, producing promising results, particularly when combined with transfer learning techniques. We observed that the outcomes of various algorithms and their combinations also rely on the quantity and type of data utilized for training. The findings suggest that employing multiple combinations of different neural networks with convolutional layers contributes to the development of a more precise system for early diagnosis of sleep apnea.

## Introduction

Sleep apnea affects millions of people around the world. It is a serious disorder in which breathing is repeatedly interrupted long enough to disturb sleep. This interruption usually causes a decrease in oxygen levels and an increase in blood pressure. carbon dioxide levels in the blood [1]. There are two main forms of sleep apnea: obstructive and central [1].

Obstructive sleep apnea (OSA): This is the most common type of sleep apnea, which occurs when the muscles in the throat relax and block the flow of air to the lungs [2]. It can have several negative health consequences such as fatigue and daytime sleepiness, concentration and memory problems, high blood pressure and heart problems [3].

Central sleep apnea (CSA): occurs when the brain does not send correct signals to the muscles that control breathing. This condition is less common than OSA. Some of the consequences of CSA may include chronic fatigue and morning headache [4].

One of the most notable challenges in sleep apnea research is the difficulty in diagnosing it. Often, patients do not have clear, specific symptoms that allow the condition to be easily identified. This lack of clarity in symptoms can delay diagnosis and, consequently, increase the risk of health complications for those affected [5]. Another major challenge lies in the variability in the definition and classification of sleep apnea. Different studies and organizations may use different criteria to define and classify this disorder.

Failure to detect sleep apnea early is a major problem that can have serious long-term health consequences. It is essential that people are informed about the symptoms of sleep apnea and seek medical attention if they experience any of them [6]. Additionally, healthcare professionals should be aware of the signs and symptoms of sleep apnea and consider diagnostic testing if there is a suspicion that a patient may have this disorder. Early diagnosis and appropriate treatment can significantly improve the quality of life of people with sleep apnea and reduce the risk of serious complications.

Currently the diagnosis is made using the techniques of conventional Polysomnography (PSG), Respiratory Polygraphy (RP) and Simplified Respiratory Polygraphy (PRS) [7].

PSG consists of the simultaneous recording of neurophysiological and respiratory variables that allow us to evaluate the quantity and quality of sleep, as well as the identification of different respiratory events and their respiratory and neurophysiological impact. PSG is usually performed in a sleep unit [8]. However, they can also be done at home in many cases, where, in addition to breathing, oxygenation, sleep posture, cardiac function, brain and muscle activity are measured while the subject sleeps [9]. This test, although risk-free, is not free of complexity, requires the use of technologically sophisticated equipment, and requires the intervention of a specialized sleep technician. Although PSG is the most precise method, its limited availability, the enormous target population, the costs, and its complexity, make this technique a poor “gold standard.”

RP consists of the analysis of respiratory variables without evaluating neurophysiological variables and is an accepted technique as a diagnostic approach for SAHS [10]. Today, RP is considered an alternative to PSG that can be used both in the sleep laboratory and at the patient’s home.

Validation studies of PRS equipment for the diagnosis of SAHS have been very promising. These devices have a smaller number of channels than PR, consisting of two main channels (oronasal flow measurement and skin oximetry) and usually incorporate some secondary channels such as snoring, heart rate, body position and/or ventilatory effort [11].

The use of this simplified equipment will not only reduce the costs of sleep studies but will also greatly favor diagnostic accessibility to large masses of the population for whom it is not possible now [5]. Furthermore, in the follow-up of patients with SAHS, both treated and untreated, if the evolution is not satisfactory due to the reappearance of symptoms, the presence of side effects derived from the treatment or the appearance of associated complications, they must be sent again. to a Sleep Unit for evaluation, diagnosis and treatment if appropriate.

This survey primarily focuses on identifying the latest artificial intelligence techniques, wearable devices, types of data, and preprocessing methods employed in the diagnosis of sleep apnea. A detailed analysis of the 28 studies included in Table 4 is presented. These studies explore diverse types of physiological data such as ECG, PPG, SpO2 signals, accelerometry, and respiratory rate, which are collected using a variety of wearable devices, including wristbands, adhesive patches, prototype chest sensors, and inertial devices. The preprocessing techniques applied to these data vary considerably and are designed to improve the quality of the signals prior to analysis. These techniques include data segmentation, feature extraction, filtering, peak detection, noise removal, normalization, and artifact handling, tailored to the specific characteristics of each signal type and device. Furthermore, some research implements advanced strategies such as data interpolation and event detection from temporal patterns in the signals. This comprehensive approach to data collection and preliminary processing is crucial to optimize the performance of AI models that are subsequently used to detect and classify sleep apnea events. This demonstrates how the combination of advanced devices, specific preprocessing techniques, and the integration of AI are essential for advancing the accurate detection of sleep apnea.

The predominant AI models in studies include convolutional neural networks (CNNs) and recurrent neural networks (RNNs), particularly LSTMs, which are the most widely used in sleep apnea detection. Furthermore, the combination of these architectures with transfer learning models has shown promise for early diagnosis. Traditional algorithms, such as Random Forest, are also frequently used.

However, model performance is closely linked to the quantity and quality of the data used for training, demonstrating the importance of employing a variety of neural networks and convolutional layers to improve accuracy. Despite advances, limitations remain, such as the variability in the definition and classification of sleep apnea across studies, as well as the limited accuracy of some devices in measuring all sleep stages.

The general objective of the project is to develop an intelligent tool that allows identifying and measuring new variables during sleep apnea and associating them with oxygen saturation data, allowing the design of a diagnostic method sleep apnea device that complies with current regulations. The working hypothesis is based on that an AI algorithm trained with a clinical database of sleep apnea patients will allow a robust diagnosis of this disease through measurements made with new wearable medical devices.

## Materials and Methods

The main goal of our systematic review is to identify current challenges that sleep apnea syndrome diagnoses and wearable devices must deal with in order to apply artificial intelligence models. In Table 1, the research questions and objectives that are addressed throughout this study are shown.

**Table 1:** Research question

	Question	Purpose
Q1	What are the most prevalent wearables used to diagnose sleep apnea syndrome?	To determine which wearable devices have been selected to detect apnea.
Q2	What data types are used to diagnose with artificial intelligence?	To identify which type of data or signal is used to diagnose apnea
Q3	Which are the pre-processing techniques used?	To identify which pre-preprocessing techniques are used with the different data.
Q4	Which are the Machine Learning and Deep Learning models have been used?	To identify which models are the most common and which have the best results.

PRISMA guidelines were followed to select articles from the systematic review applying 4 steps: Identification, filtering, eligibility, and inclusion.

### Data Selection

WoS, IEEE, Scopus and PubMed databases were used to do the information search. The publication date was limited from January 1st 2020 to March 25th, 2024.

#### Identification

The primary objective was to compile articles encompassing terms associated with apnea detection utilizing wearable devices and artificial intelligence, utilizing medical and technical keywords identified in our search strategy outlined in Table 2. The search employed a filter targeting the abstract, title, and keywords. A total of 265 records were identified based on the search strategy across the four proposed databases.

**Table 2:** Search strategy

Source	Keywords	Records
IEEE	(“All Metadata”: “sleep apnea”) AND (“All Metadata”: “artificial intelligence” OR “All Metadata”: “deep learning” OR “All Metadata”: “machine learning”) AND (“All Metadata”: “wearable” OR “All Metadata”: ”wearables” OR “All Metadata”: “portable device” OR “All Metadata”: ”portable devices”)	41
Scopus	TITLE-ABS-KEY (“sleep apnea”) AND TITLE-ABS-KEY (“artificial intelligence” OR “deep learning” OR “machine learning”) AND TITLE-ABS-KEY (“wearable” OR ”wearables” OR ”portable device” OR ”portable devices”)	105
WoS	Topic (“sleep apnea”) AND Topic (“artificial intelligence” OR “deep learning” OR “machine learning”) AND Topic (“wearable” OR ”wearables” OR ”portable device” OR ”portable devices”)	74
PubMed	((“sleep apnea”) AND (“artificial intelligence” OR “deep learning” OR “machine learning”)) AND (“wearable” OR “portable device” OR “wearables“ OR “portable devices”)	45
Total records		265

#### Screening

The screening process involved applying the first filter with the aim of removing duplicate records with Mendeley checking the duplicates filter. 135 texts were repeated in total among four databases.

#### Eligibility

This phase was completed to select original research that includes the use of wearable devices and artificial intelligence models to detect sleep apnea. Therefore, at this point, the selected text describes the preparation process, which was carried out in two stages.

In the first stage, a revision of the titles was performed to exclude studies that did not meet the criteria: articles not written in English, opinion articles, or those lacking content based on apnea detection techniques. After this step, 53 articles were discarded, resulting in a total of 77 remaining.

In the second stage, the abstract selection process primarily focused on determining their compliance with the eligibility criteria. The criteria for this stage included:

Studies involving the use of portable devices to generate data for apnea detection and/or validation of artificial intelligence models.

Artificial intelligence techniques applicable to apnea detection using data from portable devices.

Following the application of these eligibility criteria, 28 texts were excluded. This process led to the remaining 44 articles being read in their entirety.

**Table 3:** Inclusion and exclusion criteria

Criteria	
Inclusion	Exclusion
Complete documents Publication in journals or conferences Title and abstract with the keywords used in the search	Studies not written in the English language. Publications such as Book Chapters, Survey Papers, reviews, etc. Unavailable records

#### Inclusion

**Fig. 1:**
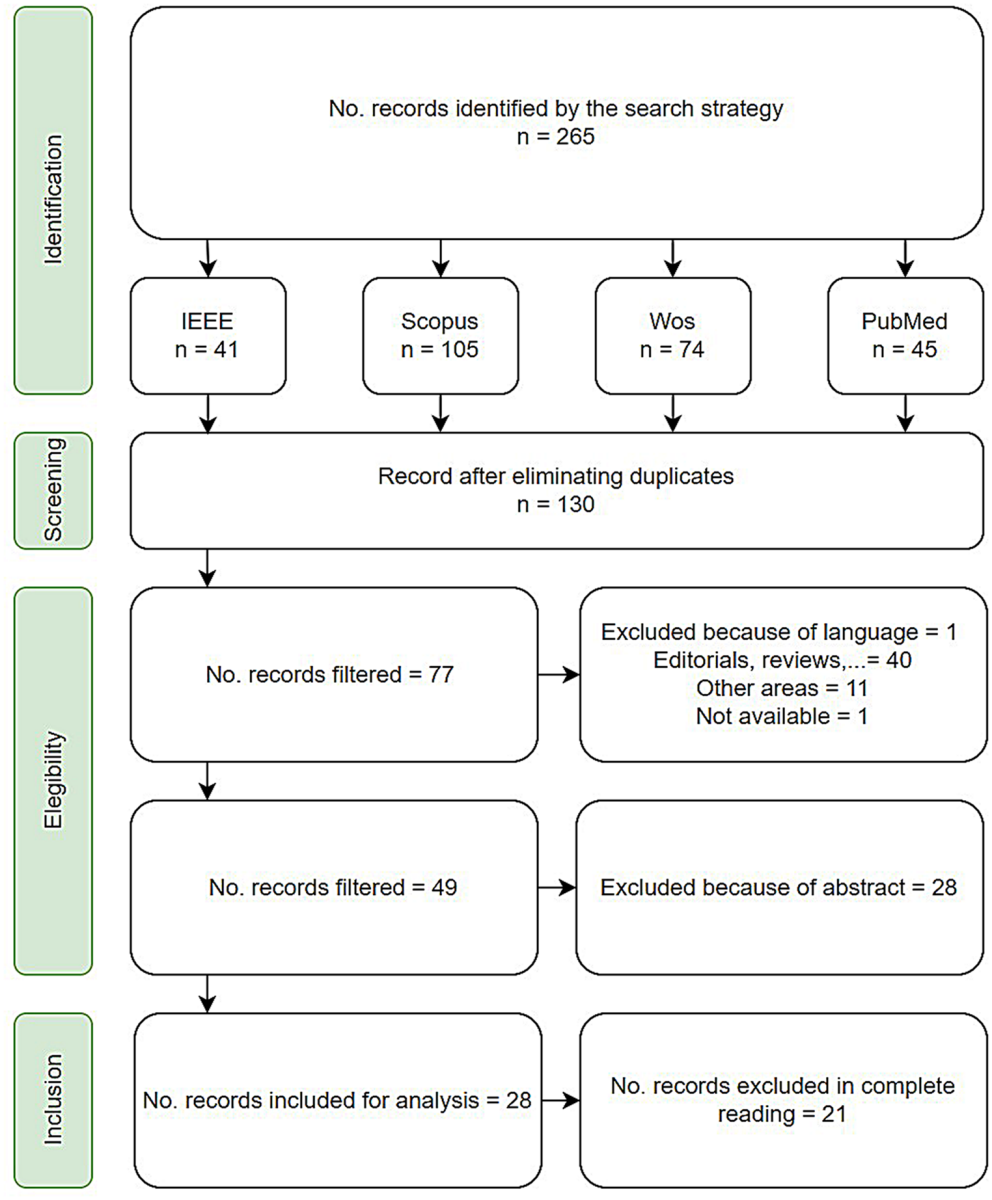
Flow chart of systematic review

Based on the outcomes of the two preceding phases, the criteria for screening records, including both inclusion and exclusion criteria, can be outlined in Table 3. After thoroughly reviewing the 49 documents that remained, 28 were subsequently chosen for inclusion in the study, Fig. 1 shows the flow chart of the systematic review. In Table 4 are shown the title and information of each included document.

**Table 4:** Review studies included and their characteristics

Title	Year	Country	Wearable device	Data type	Preprocessing techniques	AI model
Multitask Residual Shrinkage Convolutional Neural Network for Sleep Apnea Detection Based on Wearable Bracelet Photoplethysmography [12]	2022	China	PPG bracelet device	photoplethysmography (PPG)	Division in segments, peak extraction and intervals	Convolutional Neural Network: D-MMResSNet model
Airline Point-of-Care System on Seat Belt for Hybrid Physiological Signal Monitoring [13]	2022	China/Singapore	Flexible dry ECG electrode band and breathing sensor	Electrocardiogram (ECG) and breathing patterns	Feature extraction	Recurrent neural network (LSTM)
Efficient Deep Learning Based Hybrid Model to Detect Obstructive Sleep Apnea [14]	2023	India/Norway	ECG sleepify prototype	Electrocardiogram (ECG)	Division in segments	Recurrent neural network (LSTM)
A Multi-Sensor Based Automatic Sleep Apnea Detection System for Adults Using Neural Network Inference on FPGA [15]	2022	USA	ECG adhesive path and pulse oximeter	ECG and SpO2 signals	Data normalization and Scaling	Feedforward Neural Network (FNN)
Portable Detection of Apnea and Hypopnea Events Using Bio-Impedance of the Chest and Deep Learning [16]	2020	Belgium	Robin: BioZ, ECG and acceleration PSG belt	ECG, acceleration and respiratory measurements	Epoch creation	Recurrent neural network (LSTM)
Obstructive Sleep Apnoea Syndrome Screening Through Wrist-Worn Smart Bands: A Machine-Learning Approach [17]	2023	Italy	Fitbit Charge 4 and Fitbit AltaHR	photoplethysmography data	Feature extraction	Machine learning: multi-layer perceptron (MLP) classifier and random forest
At-home wireless sleep monitoring patches for the clinical assessment of sleep quality and sleep apnea [18]	2023	USA	Wearable small patches prototype	EEG, EOG, and EMG	Bandpass filter, epoch segmentation	Convolutional neural networks (CNN)
Wearable Sensor-Based Human Exhalation Rhythm Recognition using Deep Learning Neural network [19]	2022	India	comfy skin-worn motion detector prototype	Acceleration and rotational motion	Segmentation and label events	One-dimensional convolution neural network (1D-CNN)
A Wearable Device Integrated with Deep Learning-Based Algorithms for the Analysis of Breath Patterns [20]	2023	Turkey	Wearable chest accelerometer prototype with temperature	Acceleration and temperature	Noise removal	Deep learning using YOLO architecture
Classification and detection of breathing patterns with wearable sensors and deep learning [21]	2020	USA	Comfortable skin-worn inertial sensors	Acceleration and rotational motion	Segmentation	Convolution neural network (1D-CNN)
SLEEP-SEE-THROUGH: Explainable Deep Learning for Sleep Event Detection and Quantification from Wearable Somnography [22]	2023	Italy	Chest-Worn Sensor	Breathing rhythm, chest effort, body position, SpO2 and mechanical vibrations	Filtering, standardization, and signal labelling	Deep network
Machine Learning Assisted Wearable Wireless Device for Sleep Apnea Syndrome Diagnosis [23]	2023	China	Bluetooth wearable PPG and SpO2 sensor prototype ring	PPG and SpO2	Feature extraction	Machine learning
Long-Term Sleep Respiratory Monitoring by Dual-Channel Flexible Wearable System and Deep Learning-Aided Analysis [24]	2022	China	Bluetooth respiratory sensor prototype	Breathing rhythm	Filtering	1D-CNN
Sleep Apnea Severity Estimation from Tracheal Movements Using a Deep Learning Model [25]	2020	Canada	Wearable small patches prototype	Respiratory inductance plethysmography, acceleration, airflow by nasal pressure cannula, and peripheral oxyhemoglobin saturation (SpO2) by pulse oximetry.	Segmentation, labelling and feature extraction	Combination of convolutional neural network, Long Short-Term Memory (LSTM) layer, and fully connected layer
Robust Method for Screening Sleep Apnea with Single-Lead ECG Using Deep Residual Network: Evaluation with Open Database and Patch-Type Wearable Device Data [26]	2020	South Korea	T-REX TR100A patch device	ECG	Filtering, peak detection and transformation	Residual neural network (ResNet): ResNet18, ResNet34 and Resnet50
Wearable monitoring of sleep-disordered breathing: estimation of the apnea–hypopnea index using wrist-worn reflective photoplethysmography [27]	2020	The Netherlands	Wrist-worn device	PPG, respiratory activity, acceleration and HRV	Feature extraction	Convolutional neural networks (CNN)
Respiratory Event Detection during Sleep Using Electrocardiogram and Respiratory Related Signals: Using Polysomnogram and Patch-Type Wearable Device Data [28]	2022	South Korea	Polysomnogram and Patch-Type Wearable Device	ECG, acceleration	Filtering, peak detection and feature extraction	LDA, QDA, RF, MLP, SVM
Real-Time Sleep Apnea Diagnosis Method Using Wearable Device without External Sensors [29]	2020	Republic of Korea	Wrist-worn device	Acceleration, heart rate, SpO2 and respiration	Labeling	Gaussian Naive Bayes (GNB), Artificial Neural Network (ANN) and K-Nearest Neighbor (KNN)
Belun Ring (Belun Sleep System BLS-100): Deep learning-facilitated wearable enables obstructive sleep apnea detection, apnea severity categorization, and sleep stage classification in patients suspected of obstructive sleep apnea [30]	2023	China/USA	Belun ring	SpO2, pulse rate, and accelerometry signals	Feature extraction, segmentation	CNN
At-home wireless monitoring of acute hemodynamic disturbances to detect sleep apnea and sleep stages via a soft sternal patch [31]	2021	USA	skin-contact mechanics of a soft sternal patch	ECG, PPG, SCG and ACC	Filtering, normalization	RCNN
Bare-bones based honey badger algorithm of CNN for Sleep Apnea detection [32]	2024	United Emirates Arab	Soft sternal patch	ECG	Identification of R-peaks, Extraction of RR Intervals and Amplitudes, Filtering and cubic interpolation	CNN (BBHAB3 method)
Energy-Efficient Sleep Apnea Detection Using a Hyperdimensional Computing Framework Based on Wearable Bracelet Photoplethysmography [33]	2024	China	Wrist-worn device	photoplethysmography data	Segmentation, filtering, peak detection	hyperdimensional computing (HDC)
A deep transfer learning approach for sleep stage classification and sleep apnea detection using wrist-worn consumer sleep technologies [34]	2024	USA/Denmark	Wrist-worn device	Accelerometer and photoplethysmography data	Interpolation, filtering, denoising, normalization, data loss handling	DNN
Deep Transfer Learning for Automated Single-Lead EEG Sleep Staging with Channel and Population Mismatches [35]	2023	Netherlands	Wearable EEG	EEG raw data	Filtering, artifact removal, normalization, feature extraction, segmentation	CNN and LSTM with TinySleepNet deep learning
Multiscale Bidirectional Temporal Convolutional Network for Sleep Apnea Detection Based on Wearable Photoplethysmography Bracelet [36]	2023	China	Wrist-worn device	photoplethysmography data	Noise removal, peak-to-peak interval extraction, data interpolation, temporal dependence analysis	1D-MsBiTCNet model
Multiscale entropy analysis of single lead ECG and ECG derived respiration for AI based prediction of sleep apnea events [37]	2024	India	Wearable ECG	ECG raw data	Segmentation, denoising, R peak detection	Machine learning models
SomnNET: An SpO2 Based Deep Learning Network for Sleep Apnea Detection in Smartwatches [38]	2021	Ireland	Smartwatches	Spo2	Feature extraction	1D-CNN
ApneaDetector: Detecting Sleep Apnea with Smartwatches [39]	2021	USA	Smartwatches	Accelerometer	Denoising, calibration, feature extraction	Random forest

## Results and Discussion

This systematic review encompasses 28 studies retrieved from four distinct databases. Utilizing specific keywords, we identified pertinent studies published exclusively between 2020 and 2024, with six conducted in 2020, three in 2021, six in 2022, nine in 2023, and four in 2024. Throughout the review process, we identified elements providing relevant responses to the re-search questions through meticulous document analysis.

### Answer to RQ1

**Fig. 2:**
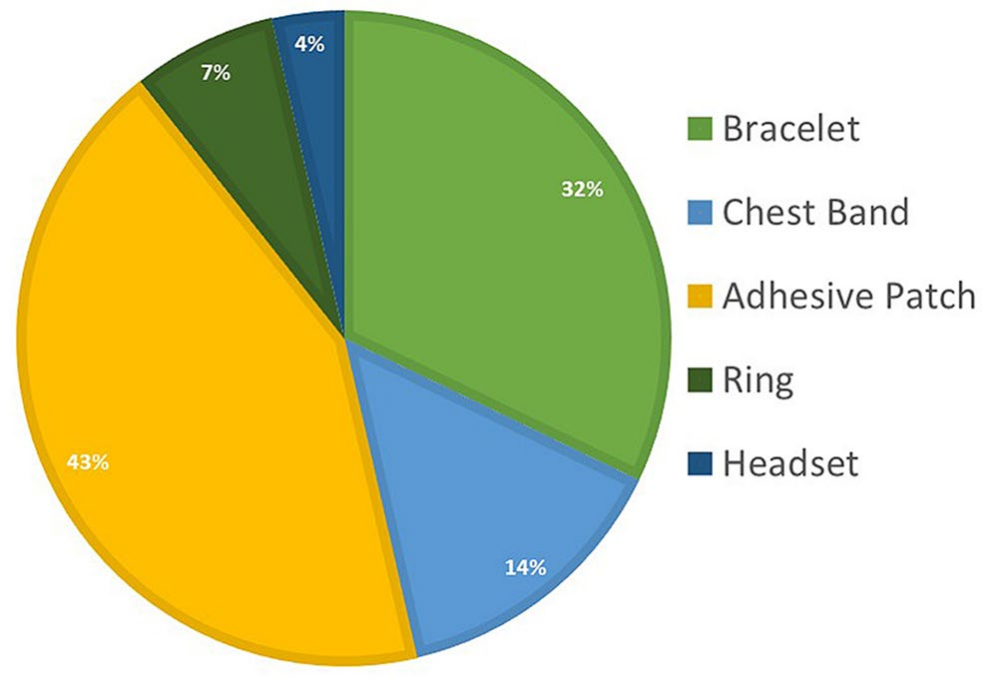
Distribution of wearable devices

The first research question centered on the types of wearable devices employed for apnea detection techniques to capture patterns associated with the disease. The identified wearable devices can be classified into four groups based on their type and location: bracelet, chest band, adhesive patch, headset, and ring. These devices are accessible commercially or are specifically designed for case study. Figure 2 illustrates the distribution of these diverse devices.

#### Bracelet

**Fig. 3:**
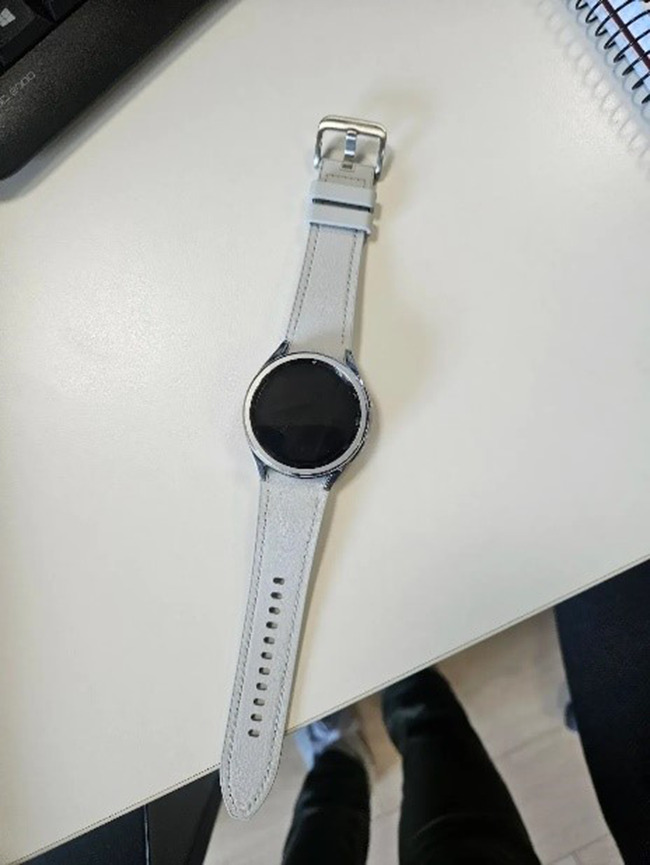
Samsung Watch 6, example of a bracelet

Bracelets offer a convenient and non-intrusive method for diagnosing sleep apnea. They eliminate the need to attach multiple sensors to the body, as required by traditional methods like polysomnography Fig. 3.

In [12], Qi Shen et al. used a wearable smart bracelet to collect data monitoring the sleep of 92 subjects. In the final development stage, they connected the device to a Raspberry Pi via Bluetooth to process the data in real-time. The bracelet was connected to the wrist and the data collected were RR intervals of ECG signal. Davide Benedetti et al. [17], used two different commercial smart bands, Fitbit Charge 4 and Fitbit Alta HR, to collect data from 78 subjects. In this process, they employed the Fitbit algorithm to conduct a preliminary examination of the sleep stages and then exported the data from the platform via API. Smart bands worn on the wrist ensure skin contact for sensors that collect acceleration measurements and photoplethysmography data. Sleep parameters derived include sleep efficiency, total sleep time, sleep fragmentation index, wake time after sleep onset, number of awakenings, and average awakening duration. Gabriele B. Papini et al. [27], studied the utilization of wrist-worn bracelet PPG devices for monitoring sleep disorders and estimation of the apnea-hypopnea index. They used one developed by Philips for research purposes. Jeon et al. [29], proposed a system for diagnosing and predicting sleep apnea using wearable devices. The study proposes a system for diagnosing and predicting sleep apnea using only a wearable device, such as the Sleep Care Kit (SCK), which measures respiration, oxygen saturation, heart rate, and 3-axis acceleration. The device is worn on the wrist, ensuring skin contact for its sensors to collect this data. The correlation between heart rate and 3-axis acceleration is analysed to predict sleep apnea after an initial learning period.

#### Chest Band

**Fig. 4:**
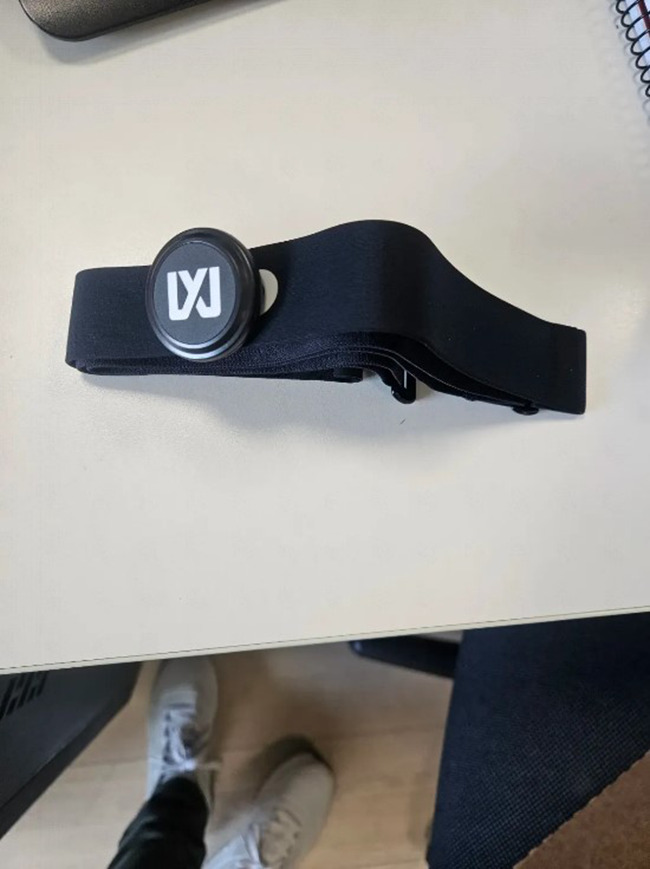
Chest Band Example

Chest bands excel in delivering both comfort and a secure fit, ensuring consistent contact with the skin for reliable signal acquisition, example in Fig. 4. Notably, their washable and reusable nature makes them a financially prudent choice for extended monitoring periods. Furthermore, their seamless integration into clothing enhances their appeal, offering a convenient and inconspicuous solution for wearable monitoring.

In [13], Ji et al. developed a chest band integrated into airplane seat belts to monitor sleep and passenger health during flights. The system combines hybrid signal detection for ECG, respiration, and motion, incorporating flexible ECG electrodes and piezoelectric belts into an elastic chest strap, along with a 3-axis acceleration sensor. Skin contact is vital for the detection of physiological signals, enabling the capture of ECG features, respiration patterns, and body position. The device provides real-time health monitoring, with a specific focus on identifying sleep apnea-hypopnea syndrome (SAHS). By seamlessly integrating wearable technology into seat belts, this innovative approach enhances passenger health monitoring and comfort during flights, paving the way for smarter in-flight care solutions. Tarim et al. [20], developed a wearable device to analyse breath patterns and tested it with three healthy volunteers. Subsequently, an additional 10 healthy volunteers were involved in the testing phase. The device includes a high-resolution triple-axis accelerometer sensor positioned on the diaphragm to detect diaphragm movements, along with a microcontroller responsible for processing and transmitting sensor data. To validate the breath pattern, a temperature sensor was placed near the nostrils on the upper lip during the measurements.

In [33], Chen et al. conducted a study involving a total of 100 subjects who willingly participated. The study focused on utilizing wearable bracelet PPG devices for sleep apnea detection. The wearable device utilized in the study served multiple functions, including data collection, synchronization, energy efficiency, deployment, and long-term monitoring. Participants wore these wearable bracelets during sleep monitoring sessions to capture PPG signals, which were synchronized with PSG data for accurate analysis. The proposed method aimed to optimize energy efficiency by reducing memory footprint, latency, and energy consumption of these devices, facilitating their deployment on systems with limited computational resources. Moreover, the study envisioned leveraging these energy-efficient methods for long-term home-based monitoring of sleep apnea, thus enhancing consistent patient care.

In [34], Olsen et al. employed wrist-worn consumer sleep technologies (CST) to gather raw data for the deep transfer learning approach utilized in sleep stage classification and sleep apnea detection. These devices were outfitted with accelerometers and photoplethysmography sensors to capture accelerometer and photoplethysmography data for analysis, involving patients with sleep-related breathing disorders, particularly concentrating on obstructive sleep apnea (OSA). It is probable that the patients included in the study underwent sleep monitoring using both clinical equipment and wrist-worn consumer devices.

In [36], Zou et al. employed commercial wearable PPG bracelets to gather data on parameters such as heart rate and blood volume changes during sleep. Using this collected data, they compiled a dataset comprising information from 92 patients. The participants in the study likely exhibited diverse sleep patterns and may have had underlying sleep apnea conditions. In [38], Arlene et al. aim to develop a high-resolution apnea detection algorithm tailored for integration into smartwatches, leveraging their capability to record SpO2.

In [39], Chen et al. utilized the Huawei Watch 2 smartwatch model to gather sensor data concurrently with the standard PSG test at the Penn State Milton S. Hershey Medical Center. The study involved twenty subjects, comprising eight males and twelve females, with ages spanning from 36 to over 72 years.

#### Adhesive Patch

**Fig. 5:**
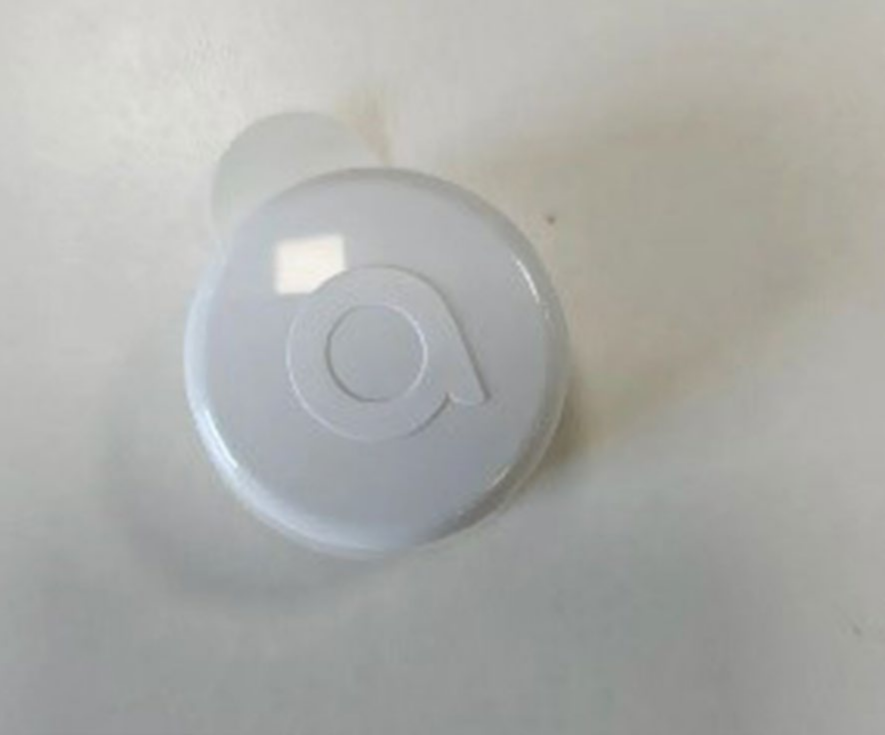
Adhesive patch Example

A thin and flexible wearable adhesive patch is crafted to be affixed directly onto the skin, facilitating continuous monitoring or targeted functionality delivery example in Fig. 5. These patches adhere comfortably to the body, ensuring inconspicuous and convenient utilization. With applications spanning consumer and medical contexts, these patches provide a non-intrusive and discreet method for monitoring health metrics or administering therapeutic substances.

In [14], Hemrajani et al., created a wearable device called Sleepify, specifically crafted to monitor ECG signals and distinguish between apnea and normal patterns. Sleepify seeks to offer a more user-friendly and easily accessible approach to capturing ECG signals while individuals are asleep, enabling patients to utilize the device in the comfort of their homes. The device incorporates ECG sensors for signal recording and employs a Raspberry Pi Pico for sleep apnea diagnosis. Hassan et al. [15], proposed a system that utilizes two types of biosensors. The system collects single channel biopotential ECG data from a chest strap and SpO2 signal from pulse oximeters. The ECG patch collects chest movement data using a single lead ECG patch.

Steenkiste et al. [16], utilized was the ROBIN wearable bio-impedance device, proficient in measuring and recording diverse bio-signals. In this context, the device recorded bioZ at the chest, providing a measurement of respiration. Skin contact is essential for the electrodes measuring ECG and bioimpedance on the chest, with the device designed to be compact and minimally intrusive. Moreover, it incorporates an accelerometer sensor to capture and quantify the dynamic movement of the patient. Kwon et al. [18], designed an integral wearable sensor with two diminutive patches, one tailored for EEG and EOG measurements on the forehead and the other for capturing EMG data on the chin. Made from flexible nanomembrane electrodes and soft, flexible materials, these patches are meticulously designed to adhere perfectly to the skin, ensuring comfort and reliable signal detection throughout the sleep cycle. Rubavathy et al. [19] employed double biocompatible epoxy, comfortable skin-worn motion detectors were affixed to the chest and abdominal aorta of everyone. These sensors were specifically programmed to capture data from a tri-axial anemometer and an angular velocity gyroscope a dual biocompatible epoxy, we applied skin-friendly motion detectors to the chest and abdominal aorta of each participant. These sensors were intricately programmed to collect data from both a tri-axial anemometer and an angular velocity gyroscope. Zhang et al. [24] developed a flexible wearable monitoring system with a functionally integrated circuit to achieve high accuracy, dual-channel functionality, and long-term sleep respiratory monitoring. The system is designed to adhere near the nostrils to capture airflow signals during sleep. It utilizes dual-channel respiratory sensors based on the principle of thermal resistance, consisting of an ultrathin polyimide (PI) tube as a flexible substrate and multiturn gold (Au) micro coils as sensitive units, all coated with a Parylene-C layer for biological insulation. Close skin contact around the nostrils is essential for detecting airflow changes, allowing the collection of simultaneous airflow signals from both nostrils, including nasal cycle detection. This innovative approach enhances respiratory monitoring accuracy while maintaining wearer comfort. Hefezi et al. [25] created a patch designed to be affixed to the user’s suprasternal notch using double-sided tape and additionally secured with clinical tape. The device incorporates a 3D accelerometer placed on the suprasternal notch to record tracheal respiratory movements, simultaneously with polysomnography. Skin contact is ensured through medical tape for accurate signal detection, enabling the measurement of tracheal respiratory movements using the accelerometer. This innovative setup provides valuable data for respiratory monitoring. Yeo et al. [26, 28] used the T-REX TR100A wearable patch device was used to record single-lead ECG data from the upper abdominal area of subjects. The patch is adhered directly to the skin of the upper abdomen, ensuring consistent skin contact for reliable signal acquisition. This method of attachment minimizes the risk of the device becoming loose or detaching, providing stable and accurate monitoring throughout its use. Zavanelli et al. [31] developed a soft sternal biopatch that records ECG, SCG, and PPG from a single location on the sternum. The supple sternal patch device comprises an ultra-thin, flexible circuit mounted on an elastomeric membrane, incorporating integrated components and nanomembrane electrodes. In their study outlined in [32], Ammar et al. proposed employing wearable patch sensors to record ECG signals for diagnosing Sleep Apnea. Through the utilization of these wearable sensors to capture ECG signals, individuals can undergo continuous monitoring of their physiological data, facilitating real-time detection of Sleep Apnea episodes. In the study by Parbat et al. [37], the objective was to develop a model for detecting sleep apnea events using physiological signals derived from ECG and ECG-derived respiration, with the aim of integrating this research into the development of AI-based IoT-connected smart wearable devices for sleep apnea detection, specifically designed as portable ECG patches.

#### Ring

**Fig. 6:**
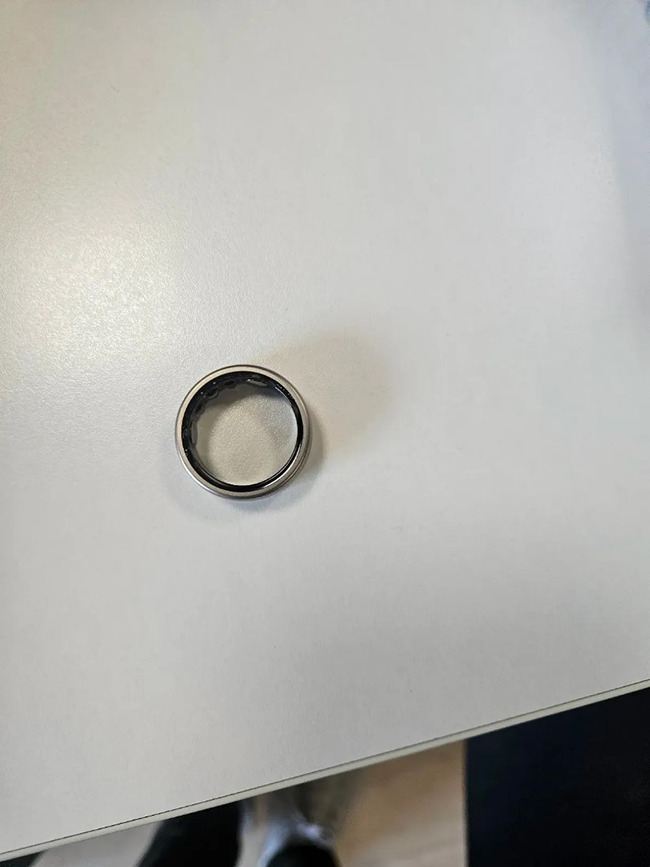
Ring Example

Smart rings, compact and discreet wearables, seamlessly integrate various sensors and technologies to gather data across different facets of well-being example in Fig. 6. Capitalizing on the thin skin around the finger, they excel particularly in heart rate readings, showcasing heightened accuracy compared to their smartwatch counterparts. As a result, these inconspicuous accessories are progressively transforming into powerful health devices.

In [23], Shaokui Wang et al. designed the circuit architecture and fabricated hardware for a Bluetooth ring prototype to collect data. The smart ring, proposed as a wearable device for SAS diagnosis, features a flexible module comprising a PPG pulse wave sensor (MAX30102), a Bluetooth transmission module (CC2640R2F), and a power supply module. All electronic components and chips are assembled on a flexible printed circuit (FPC) and housed in a 3D-printed ring. Worn on the finger, the device ensures skin contact with the PPG sensor to measure PPG signals and SpO2, with heart rate also derived from PPG signals. This compact, user-friendly design facilitates continuous and effective data collection. Zachary Strumpf et al. [30], used the commercial Belun ring [40] to collect data and developed a platform for detecting sleep apnea and analysing sleep stages.

#### Headset

**Fig. 7:**
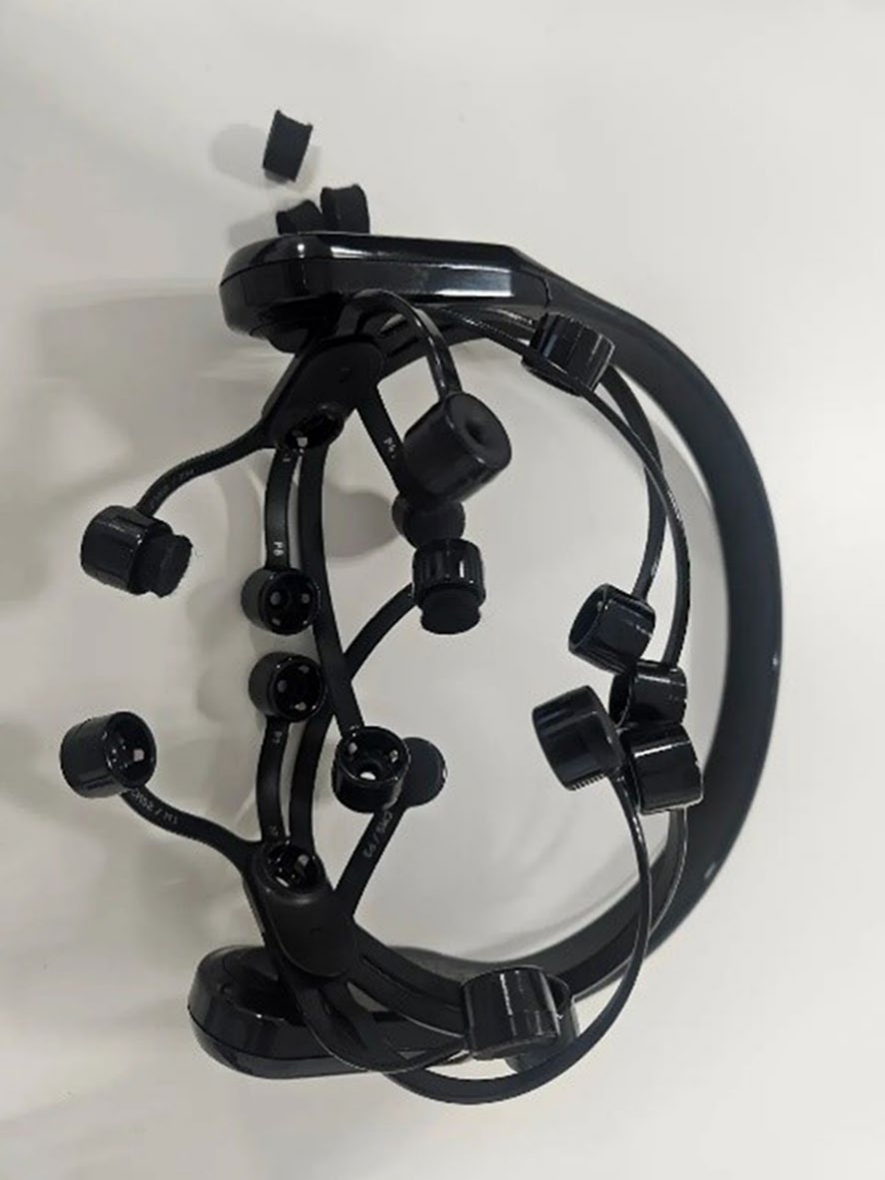
Ring Example

Compact EEG headsets, discreet yet powerful, capture precise brain activity data, transforming into essential health devices example in Fig. 7. With superior accuracy and portability, they offer early detection and monitoring of neurological conditions, revolutionizing personalized health management.

In [35], The study harnessed wearable EEG systems outfitted with dry frontopolar electrodes to streamline sleep staging processes. These advanced devices adeptly recorded single-channel EEG data throughout the sleep cycle. During data acquisition, the EEG device, featuring dry frontopolar electrodes, meticulously captured crucial brainwave signals while subjects slept.

#### Comparative Analysis of Wearables Devices

Based on our assessment, adhesive patches emerge as the most common solution in wearable devices for capturing data to characterize apnea syndrome. Integrated with sensors, adhesive patches can be affixed to a patient’s body for the continuous monitoring of vital signs, including heart rate, blood pressure, and oxygen saturation. These patches gather real-time data and wirelessly transmit it to healthcare providers, enabling remote monitoring of patients’ conditions.

Adhesive patches devices were mostly used in the analyzed studies, as they enable more than variable recording and could be attached in more than place of the body. The choice among these wearables ultimately depends on individual preferences, comfort, and the specific health metrics one seeks to monitor. Chest bands excel in accurate heart rate monitoring while they may be less comfortable for extended use or sleeping. For the case of sleep apnea, they show good results in the airplanes seats for monitoring brave patterns and ECG. Also is used with an accelerometer to monitor movements but there could potentially problems with collocation during the test. The wrist-based bracelet, for instance, has become a ubiquitous choice for daily health tracking. Their comfort and unobtrusive design make them popular among those seeking general health. Bracelets were used for sleep apnea to monitor sleep stages and control the heart rate and oxygen saturation. They could have the limitation of these type of devices is gap in performance might be found in the different kinds of information conveyed by wrist-worn smart bands and the form and type of data reception. Lastly, compact and discreet rings are gaining popularity in the wearable health tech landscape. For sleep apnea we found the use of design for the study to measure heart rate and oxygen saturation, and also commercial models that study the sleep stages. One of the studies don’t achieve precise correctly the sleep apnea in the patient.

### Answer to RQ2

The second research question delved into the data types utilized for diagnosing sleep apnea, focusing on the integration of devices and artificial intelligence models. Diagnosing sleep apnea entails scrutinizing diverse data types obtained through various methodologies, with biomedical data being a primary component. Polysomnography (PSG) stands as the gold standard, involving a comprehensive sleep study that monitors key physiological parameters, including brain waves, eye movements, heart rate, respiratory effort, and oxygen saturation. In addition to PSG, actigraphy captures movement patterns, offering insights into sleep-wake patterns and overall quality. Oximetry measures blood oxygen levels, detecting potential sleep apnea through sustained drops in saturation. Further, respiratory monitoring evaluates airflow and effort, while cardiorespiratory monitoring integrates cardiac and respiratory parameters, providing a more thorough comprehension of the diagnostic process [41].

#### Photoplethysmography Data

The photoplethysmography data have been recorded and used in [12, 17, 23, 25, 27–29, 31, 33, 34, 36]. Photoplethysmography (PPG) data is derived from a non-invasive optical technique that measures changes in blood volume in the microvascular bed, providing valuable insights into physiological parameters. This method monitors heartbeat characteristics by analyzing variations in light intensity passing through or reflected by tissue. PPG is also used to estimate oxygen saturation (SpO2) and assess autonomic nervous system variability. Easily obtained through wearable devices like bracelets, rings, watches, and fingertip pulse oximeters, PPG sensors detect red (λ = 660 nm) and infrared (IR, λ = 880 nm) light using a photodiode sensor to capture these signals.

Beyond sleep apnea detection, PPG is applied to monitor heart rate, respiratory rate, and SpO2 levels and has been explored for predicting sleep stages and evaluating the severity of sleep-related breathing disorders. In the context of sleep apnea, PPG identifies decreases in SpO2 levels during apneic events. Features extracted from PPG signals can be used to train machine learning models for apnea detection. Wearable devices leveraging PPG technology are considered effective and recommended for sleep apnea monitoring. This capability makes it a critical tool for both research and clinical applications.

#### Electrocardiography Data

Electrocardiography (ECG) records the heart’s electrical activity over a period and has been used in [13–16, 26, 31, 32, 37]. ECG signals offer valuable insights into both heart rate and heart rate variability, providing a reliable method for monitoring cardiac activity. ECG records the electrical activity of the heart, capturing P waves, QRS complexes, and T waves for each heartbeat, while also allowing the derivation of respiratory information through ECG-derived respiration (EDR). A regular ECG pattern indicates a normal cardiac cycle. This non-invasive technique is ideal for home sleep monitoring and prolonged patient observation.

ECG signals are acquired using electrodes placed on the body to detect the electrical signals generated by the heart. Single-lead ECG can be recorded using portable, non-invasive technologies, such as soft sternal patches or wearable devices, which provide wireless recording capabilities. In polysomnography (PSG), ECG is often one of the standard channels recorded. The use of patch-type wearable devices ensures convenient, continuous recording without invasive procedures.

Beyond sleep apnea detection, ECG is applied for identifying cardiac arrhythmias, assessing cardio-respiratory coupling, monitoring heart rate variability (HRV), and detecting neurological abnormalities. For sleep apnea, ECG signals are particularly valuable as apneic events are reflected in changes such as bradycardia followed by tachycardia and variations in R-R intervals. HRV shows a strong correlation with sleep apnea, while EDR can approximate respiratory patterns and frequencies. These features, coupled with machine learning models, enable accurate detection of sleep apnea, supporting a multi-modal approach when integrated with respiratory signals and accelerometer data.

#### Breathing Rhythm Data

Breathing patterns or respiratory rhythm data are crucial in the context of sleep apnea detection, and the utilization of this data is proposed in [13, 16, 22, 24, 27, 29]Facilitating early detection, the system analyzes breath patterns to identify abnormalities linked to sleep apnea-hypopnea syndrome (SAHS) in its initial phases. Respiratory rhythm refers to the cyclical variations in lung volume during breathing, ranging from a regular rhythm indicative of health to the complete absence of respiration. This signal is directly measured using nasal airflow sensors, thoracic and abdominal motion signals in polysomnography (PSG), or respiratory effort belts. It can also be indirectly acquired through chest or abdominal accelerometers, pressure signals, or ECG-derived respiration (EDR). Portable devices often measure diaphragm movement using accelerometers.

Beyond detecting sleep apnea, respiratory rhythm analysis is utilized to assess respiratory function, monitor various medical conditions, and evaluate the likelihood of successful weaning from mechanical ventilation. In sleep apnea detection, apnea is marked by the cessation of airflow, while hypopnea represents a reduction in breathing amplitude. Changes in respiratory rhythm and effort detected by various sensors are crucial for identifying apnea events and classifying their type (obstructive or central). Abnormal breathing patterns, such as apnea, hypopnea, and normal respiration, can be analyzed using portable sensors and deep learning for comprehensive and accurate diagnosis.

#### Oxygen Saturation Data

An additional category of data utilized is oxygen saturation, commonly referred to as SpO2 in the literature. This information is critical for evaluating sleep apnea and is frequently combined with other metrics like the apnea-hypopnea index (AHI) to assess the severity of sleep apnea and its effect on oxygen levels during sleep. This inclusion is suggested in [15, 22, 23, 25, 29, 30, 38] as it aids in the detection of episodes of oxygen desaturation that may occur during sleep apnea. It is typically acquired using pulse oximeters attached to the finger, which measure the absorption of red and infrared light by the blood. Portable devices, such as wristbands, chest straps, and soft sternal patches, also monitor SpO2. This signal has applications beyond sleep apnea detection, including monitoring respiratory conditions, assessing the need for supplemental oxygen, and detecting hypoxemia in clinical settings.

In the context of sleep apnea, SpO2 plays a vital role in identifying episodes of oxygen desaturation that occur during apneic and hypopneic events. These desaturation episodes are reflected in reduced blood oxygen levels and are key for diagnosing and determining the severity of sleep apnea. Parameters such as the oxygen desaturation index (ODI) and time spent below specific SpO2 thresholds are extracted to assess sleep apnea’s impact. The inclusion of SpO2 data enables a comprehensive evaluation of the condition, as it highlights potential health consequences like cardiovascular disease, stroke, and cognitive impairment.

#### Body Position and Tracheal Movements Data

Accelerometers offer the capability to gauge body position and respiratory movements, including tracheal movements, as suggested in [16, 19–22, 25, 27–31, 34, 39]. The authors propose the utilization of accelerometers to monitor body position during sleep, a critical factor in assessing the influence of sleeping positions—such as supine, prone, left lateral, or right lateral—on the severity of sleep apnea. Accelerometers detect changes in gravitational forces across different axes when integrated into portable devices like smartwatches or chest patches, providing essential data for activity tracking, fall detection, and sleep pattern monitoring. Additionally, accelerometers can capture respiratory movements, detecting vibrations and motion near the trachea or upper body during breathing. This capability allows the identification of abnormal tracheal patterns, such as reduced motion or pauses, which may indicate apnea or hypopnea events.

Body position monitoring is particularly useful in addressing positional sleep apnea, which worsens in certain postures (e.g., supine). By understanding and managing positional apnea through accelerometer data, a targeted approach to treatment becomes feasible. The integration of wearable sensors such as patches, bracelets, and rings enhance the convenience of at-home monitoring, providing a comprehensive view of body orientation and respiratory movement. Features like tracheal motion amount and respiratory effort cycles can further aid in estimating sleep apnea severity, enabling precise detection and personalized management of the condition.

#### Rotation Motion Data

In addition to examining body movement, the consideration of rotational movements, as suggested in [19, 21] using a gyroscope, could be incorporated. Within the realm of wearable sensors designed for respiratory analysis, a gyroscope offers insights into the angular movement of the chest and abdominal area around an axis, measured as angular velocity or changes in orientation during breathing. This integration proves particularly advantageous as it provides valuable data regarding both the movement and orientation of the device and the specific body part to which it is attached. Gyroscopes are often combined with accelerometers in inertial measurement units (IMUs) within wearable sensors placed on the chest or abdomen for enhanced respiratory monitoring.

Beyond respiratory analysis, gyroscope signals find applications in activity recognition, gesture detection, and biomechanical analysis. In the context of sleep apnea, rotational movements of the chest and abdomen detected by gyroscopes can reveal breathing patterns and disruptions associated with apnea or hypopnea events. Such movements may correlate with respiratory effort and airway obstruction, making gyroscopes an essential tool for identifying and characterizing sleep apnea-related respiratory abnormalities.

#### Mechanical Vibrations Data

In [22, 31], the utilization of mechanical vibration data, such as seism cardiography (SCG) vibrations, was also suggested. This method necessitates optimal conformal contact between the device and the skin for efficient signal transduction, like the requirements for patch devices. It leverages physical oscillations or vibrations produced by the body, such as those caused by respiration, snoring, or cardiac activity, to analyze mechanical heart performance and enable precise differentiation between various heartbeat classes. These vibrations are captured using sensors like accelerometers, seism cardiography (SCG) sensors, or acoustic sensors (microphones), with SCG specifically detecting cardiac vibrations propagating through the chest.

Beyond cardiac function monitoring, this signal is also used to detect body movements and analyze respiratory sounds like snoring. In the context of sleep apnea detection, snoring is a common symptom of obstructive sleep apnea, and changes in respiratory effort during apneic events can alter the mechanical vibrations detected in the chest. SCG can reveal changes in cardiac mechanics related to apnea-induced stress, making it a valuable tool for diagnosing and understanding the condition.

#### Temperature Data

In [20], the proposal involves utilizing temperature data to validate breath patterns by placing a sensor on the upper lip near the nostrils during measurements. This positioning allows for the identification of hot and cold air flows during exhaling and inhaling, correlating nasal breathing with diaphragm movements. Temperature data typically represents body temperature measured at the skin surface and is acquired through sensors such as thermistors or infrared sensors integrated into wearable devices like rings or patches. Additionally, nasal airflow temperature can be utilized to confirm breathing patterns.

Beyond respiratory validation, temperature monitoring is applied to track sleep-wake cycles, detect fever, and observe physiological responses. While not a primary signal for direct apnea event detection, changes in body temperature can indirectly reflect sleep quality and disruptions caused by apnea. Furthermore, nasal airflow temperature aids in confirming the absence of respiration during suspected apnea episodes, reinforcing its utility in sleep apnea analysis.

#### Electromyogram, Electrooculogram and Electroencephalogram Data

There is a distinctive proposal that employs a combination of different data sets. In [18], the authors advocate for the incorporation of an Electromyogram (EMG), Electrooculogram (EOG), and EEG). Integrating this diverse data in sleep monitoring yields numerous advantages for evaluating sleep quality and detecting sleep disorders. EEG data plays a crucial role in identifying distinct sleep stages, encompassing both rapid eye movement (REM) sleep and non-REM sleep, this data is also used in [35]. EOG and EMG data prove valuable in detecting sleep apnea events, with abnormalities in eye movements (EOG) and muscle activity (EMG), especially in the chin area, serving as indicators of breathing disruptions during sleep, aiding in sleep apnea diagnosis. The amalgamation of EEG, EOG, and EMG data allows for a more comprehensive assessment of sleep quality and disorders. This multi-channel approach paints a more detailed and accurate picture of an individual’s sleep patterns and potential issues.

EOG captures electrical potential near the eyes to monitor eye movements, with applications in studying ocular motion disorders and alertness levels. In PSG, EOG signals distinguish REM sleep, where apnea frequency and characteristics may differ from non-REM sleep. Spectrograms derived from EOG help identify sleep stages, making EOG indispensable for detecting REM-related apnea events.

EEG records brain activity through electrodes on the scalp, classifying its spectrum into frequency bands such as alpha, beta, gamma, delta, and theta, corresponding to different brain states. Beyond sleep apnea detection, EEG is used to diagnose neurological disorders like epilepsy, study sleep architecture, and monitor cerebral function. In PSG, EEG is the gold standard for determining sleep stages and identifying arousals critical for scoring apnea events and calculating the AHI. Changes in EEG patterns are associated with apnea events and sleep fragmentation. Single-channel EEG studies further explore its use in sleep apnea detection.

The integration of EEG, EOG, and EMG data provides a multi-channel approach, enabling a comprehensive assessment of sleep quality and disorders. This holistic approach offers detailed insights into sleep patterns and the accurate detection of abnormalities like sleep apnea.

The distribution of data types used in the included studies is given in Table 5.

**Table 5:** Distribution of data type

Data type	Percentage	References	*N*
Photoplethysmography	39%	[12, 17, 23, 25, 27–29, 31, 33, 34, 36]	11
Electrocardiography	32%	[13–16, 26, 31, 32, 37]	9
Breathing rhythm	21%	[13, 16, 22, 24, 27, 29]	6
Oxygen saturation	25%	[15, 22, 23, 25, 29, 30, 38]	7
Body position and tracheal movements	46%	[16, 19–22, 25, 27–31, 34, 39]	13
Rotation motion	7%	[19, 21]	2
Mechanical vibrations	7%	[22, 31]	2
Temperature	4%	[20]	1
Electromyogram	4%	[18]	1
Electrooculogram	4%	[18]	1
Electroencephalogram	7%	[18, 35]	2

### Answer to RQ3

The third research question centers on the utilization of preprocessing techniques for the classification and prediction of sleep apnea. Preprocessing, a pivotal factor influencing the effectiveness of any AI-based system, holds a crucial role in the removal of artefacts and noise from data, leading to enhanced image quality and improved feature extraction. It is noteworthy that a significant majority of the studies included in the analysis embraced specific preprocessing methods to refine their datasets.

#### Filtering

Filtering is a critical process aimed at purifying physiological signals like EEG, EOG, and EMG to enhance data quality for analysis. Two primary filter types are utilized. Bandpass filtering, vital in EEG for isolating sleep-related frequency components, enables focused pattern analysis. Concurrently, notch filtering is instrumental in eliminating specific frequencies, such as power line interference, reducing noise and ensuring signal accuracy. These filtering methods play a crucial role in generating clean and reliable physiological signals essential for comprehensive sleep monitoring. Notably, in the case of [20, 35], filtering was applied to remove noise from accelerometer data, while in [18], both bandpass and notch filtering were employed to eliminate noise and cutoff frequencies from EEG, EOG, and EMG signals. Additionally, in the case of [22, 24, 28, 31–33, 35, 36], diverse bandpass filters like third-order Butterworth or low pass were applied. Physiological signals, such as ECGs, are exposed to various types of noise that can interfere with their analysis. Common noise includes motion artifacts, caused by body or electrode displacements; baseline drift, slow oscillation due to breathing or changes in sensor contact; EMG, which adds high-frequency components; mains interference (50–60 Hz, depending on the region); and general high-frequency noise from electronic devices. Various filters are used to clean these signals: passband filters retain only the frequencies of interest; notch filters remove electrical interference; median and moving average filters smooth the signal and correct outliers; and wavelet-based methods or specialized algorithms like Pan-Tompkins help preserve ECG morphology. This filtering approach is also applied to other signals such as PPG, EEG, CCA, and SpO₂, each with specific characteristics and challenges. For example, accelerometer signals are particularly noisy and require filters that preserve motion peaks, such as the total variation filter. In the case of respiratory or SpO₂ signals, multiple preprocessing techniques are combined to mitigate the impact of noise without losing relevant information. In short, selecting the right filter is key and must be tailored to both the signal type and the predominant noise, as well as the analysis objective.

#### Interpolation

Interpolation preprocessing techniques are essential for handling irregular or incomplete datasets by estimating values between known data points [45]. They play a vital role in ensuring data integrity and facilitating accurate analysis and modeling across various fields, from signal processing to image analysis. In [32], cubic interpolation is utilized to standardize the time intervals between data points, ensuring a consistent set of points for both amplitudes and RR intervals across segments of ECG data. In [34], both ACC and PPG signals were interpolated to a uniform time series with a sampling rate of 32 Hz. Signals exceeding this rate underwent lowpass-filtering before down-sampling to mitigate aliasing effects. Conversely, signals with sampling rates below 32 Hz were interpolated using a piecewise cubic Hermite interpolation polynomial (PCHIP) to align with the desired sampling rate. In [36], Interpolation has been employed to standardize the data for analysis, whereby all one-minute PPI series were adjusted to a uniform length of 100 sample points using a cubic spline interpolation algorithm. This process ensured consistency in the data inputs for the deep learning model.

#### Data Normalization

Data normalization, a pivotal step in data preprocessing, is utilized to standardize or scale variable values within a dataset [43]. The primary objective is to unify diverse features onto a consistent scale, preventing any single variable from unduly influencing the analysis. Typically achieved by subtracting the mean value of the signal and dividing by the standard deviation, this process yields a normalized signal with a mean of zero and a standard deviation of one, as exemplified in [22, 26, 31, 34]. For [15], the authors implemented a normalization procedure, employing the MinMax() scaler technique from the Python sklearn library to scale values within the range of 0 to 1. Min-max normalization is a preprocessing technique used to scale feature values in a dataset to a specific range, usually between 0 and 1. Its main objective is to prevent variables with larger scales from dominating in algorithms sensitive to data magnitude, such as distance-based methods or neural networks. This type of normalization improves training efficiency, accelerates model convergence, and ensures that all features have the same relative importance. In the context of sleep apnea studies, this technique was applied to physiological signals such as SpO₂ and PPG to reduce numerical ranges and facilitate model learning. A variant that normalizes data to the range [-1, 1] is also used, particularly for accelerometer signals, centering them around zero and reducing the influence of outliers by using the median and interquartile range. In addition, there are other methods such as standard deviation-based normalization, which standardizes data based on its mean and variance, and robust normalization, which is based on statistical measures that are resistant to outliers. Ultimately, normalization is a crucial step that improves the stability and performance of machine learning models, and its choice must be tailored to the specific characteristics of the dataset and the algorithm used.

#### Segmentation-Epoch Creation

Epoch segmentation is a crucial step in sleep monitoring, involving the division of a continuous flow of biomedical data into distinct time intervals, known as epochs [42]. This segmentation facilitates the analysis and interpretation of various sleep stages and the identification of sleep disorders through the examination of physiological signal changes over time. Moreover, the adoption of epoch segmentation allows the integration of machine learning algorithms, including Convolutional Neural Networks (CNNs), for automated tasks such as sleep scoring and apnea detection. This method was implemented in nine of the studies included in the analysis [12, 14–16, 18, 19, 21, 25, 30, 32, 33, 37]. Physiological signal segmentation techniques vary depending on the signal and the analysis objective, with fixed-duration segmentations being common. One-minute segments are used for ECG and SpO2, aligned with minute apnea labels; 5-minute segments for analyzing heart rate variability (HRV); and 30-second epochs for EEG and bioimpedance signals, typical in sleep scoring. Sliding 11-second windows with overlaps (10 s) are also used for more accurate detection of second-minute apneas, and 60-second sliding windows for PPG. For tracheal motions, 150-second segments are used. Segmentation is commonly performed in contiguous or overlapping windows, aligned with temporal annotations, and may include processing of R peaks in the ECG. The chosen duration should balance the capture of relevant features with temporal resolution. Additionally, preprocessing steps such as filtering, normalization, or denoising can be applied within each segment to improve signal quality before analysis.

#### Feature Extraction

Feature extraction involves the conversion of raw data into a meaningful set of features, essential for characterizing and comprehending underlying patterns. This process entails the identification and selection of pertinent characteristics or patterns from the gathered data signals. By extracting these relevant features from data signals, the system aims to encapsulate crucial information required for precise classification and analysis through machine learning techniques. Ultimately, this contributes to the effective detection of health conditions. Notably, this approach was employed in six of the studies incorporated into the analysis [13, 17, 23, 27, 28, 30, 35, 38, 39]. Sleep apnea detection relies on a broad set of features extracted from various physiological signals, each providing specific information about apneic events. Metrics such as heart rate variability (HRV), multiscale entropy, RR intervals, and R-peak amplitude are obtained from the ECG, reflecting autonomic nervous system activity. SpO₂ provides indicators such as the delta index, statistical, frequency, and entropy characteristics, as well as measures related to the duration of hypoxemia. Spectral and nonlinear features such as banded power and Hjorth parameters are extracted from the EEG, allowing analysis of sleep stages and the effects of apnea on brain activity. The PPG signal provides information similar to HRV, but from the peripheral pulse, including entropy and pulse variability parameters. Accelerometers record body movements, allowing identification of altered patterns during apnea. Respiratory signals reveal airflow irregularities and thoracoabdominal asynchronies, while tracheal motion sensors and audio recordings capture respiratory events and sounds characteristic of apnea. This multimodal approach enables a comprehensive and robust characterization of apneic episodes during sleep.

#### Labeling

Labeling involves categorizing and identifying various respiratory events based on collected data, a pivotal step for recognizing and characterizing respiratory patterns with potential applications in clinical settings for diagnosing and monitoring breathing-related conditions. In the studies proposed by [19, 22, 25, 29], operators manually labeled time windows or analyzed created segments from a past stage. The association of sensor data with corresponding respiratory event categories is crucial for training and evaluating artificial intelligence models in detecting and classifying apnea and hypopnea events. This labeling process facilitates the development of a model capable of accurately identifying and characterizing respiratory events from sensor data, thereby contributing to the estimation of sleep apnea severity. Examples of labels used in sleep apnea classification vary depending on the study focus and available data. These include binary classifications such as “Apnea vs. Normal” or “Event vs. Non-Event,” as well as categories based on apnea severity, such as “Mild OSAS” and “Severe OSAS,” or apnea types such as “OSA” (Obstructive) and “CSA” (Central). Signal quality labels, such as “Quality Signal” vs. “Poor Quality Signal,” and breathing pattern labels, such as “Normal Breathing” vs. “Apneas” vs. “Irregular Breathing Patterns,” are also employed. Additionally, some studies include labels for arousal event prediction and selective classification, such as “Apnea,” “Normal,” and “RERA.” These labels are used depending on the study objective and the level of detail needed to diagnose sleep apnea.

#### Peak Detection

Peak detection involves identifying the local maxima or minima in a signal, with the aim of automatically pinpointing points where values significantly surpass neighboring data points, indicating distinctive features or peaks. In the realm of ECG and PPG peak detection, as applied in studies [12, 28, 32, 37], algorithms play a pivotal role in automating the localization of these peaks within the signal. Regarding ECG signals, the focus is on R-peak detection, representing the ventricular depolarization of the heart. Various methods, such as threshold-based techniques, template matching, and machine learning approaches, can be employed for accurate R-peak identification. In the [28] study, the adaptive threshold algorithm proposed by Christov et al. was utilized for R-peak detection. Additionally, in the [12]study, the PPG extraction algorithm introduced by Elgendi et al. [44] was employed to eliminate baseline drift in the PPG signal and pinpoint its systolic peak. In [33, 36] studies, Peak detection algorithms were employed to detect peaks within the PPG signal, facilitating the calculation of peak-to-peak intervals for subsequent analysis.

There are several algorithms used for spike detection in physiological signals, especially for sleep apnea. The Pan-Tompkins algorithm is common for detecting R spikes in ECG signals, using filters and an adaptive threshold. The Hamilton algorithm is also employed for R spikes in the ECG, adjusting the detection to the local maximum of the signal. The combined adaptive threshold algorithm improves the accuracy of R spike detection. Other traditional methods identify spikes through signal transitions, although they can be inaccurate during central sleep apnea events. An improved algorithm addresses this by adjusting amplitude and interspike distance thresholds. In addition, techniques are used to detect spikes in the respiratory signal derived from ECG or accelerometers, and statistical analysis for apnea events. Peak detection is also applied to PPG signals using an iterative process and binary wavelet, and for QRS complexes in the ECG, adaptive filters and techniques are used to improve the accuracy of R and QRS peak detection.

The distribution of preprocessing techniques used in the included studies is given in Table 6.

**Table 6:** Distribution of preprocessing techniques

Preprocessing techniques	Percentage	References	*N*
Segmentation-epoch creation	28%	[12, 14–16, 18, 19, 21, 25, 30, 32, 33, 37]	9
Feature extraction	19%	[13, 17, 23, 27, 28, 30]	6
Data Normalization	13%	[15, 22, 26, 31]	4
Filtering	19%	[18, 20, 22, 24, 28, 31]	6
Labelling	13%	[19, 22, 25, 29]	4
Peak detection	6%	[12, 28]	2
Interpolation	3%	[32]	1

### Answer to RQ4

The fourth research question delves into the exploration of prevalent machine learning techniques and neural network architectures utilized in the classification and prediction of sleep apnea. While machine learning encompasses a broad spectrum of algorithms and methodologies for data analysis and pattern recognition, neural networks, a subset within this domain, stand out for their ability to mimic the human brain’s neural structure. In this context, understanding the diverse array of machine learning models and neural network frameworks becomes paramount for addressing the complexities inherent in diagnosing and predicting sleep apnea.

#### Machine Learning

This section addresses RQ4, which examines various machine learning models and their corresponding achievements. Table 7 presents a comprehensive overview of the distribution of different methods and metrics utilized in the analysis.

**Table 7:** Distribution of machine learning methods and metrics

Machine Learning models	Percentage	References	Metric	*N*
Random forest	28%	[17, 23, 28, 37, 39]	accuracy, specificity, sensitivity and F1	5
Linear Discriminant Analysis	6%	[28]	Accuracy, specificity, sensitivity, F1 and Cohen’s kappa	1
Quadratic Discriminant Analysis	6%	[28]	Accuracy, specificity, sensitivity and F1	1
Support vector machine	17%	[23, 28, 37]	Accuracy, specificity, sensitivity and F1	3
Gaussian Naive Bayes	6%	[29]	Accuracy, precision, recall and F1	1
K-nearest neighbors	11%	[23, 29]	Accuracy, specificity and sensitivity	2
XGboost	6%	[23]	accuracy, specificity, and sensitivity	1
Decision Tree	6%	[37]	Accuracy, specificity, sensitivity, AUC and False Positive Rate	1
Adaptive Decision Tree	6%	[37]	Accuracy, specificity, sensitivity, AUC and False Positive Rate	1
Voting Classifier	6%	[37]	Accuracy, specificity, sensitivity, AUC and False Positive Rate	1
Stacked Classifier	6%	[37]	Accuracy, specificity, sensitivity, AUC and False Positive Rate	1

In five studies [17, 23, 28] proposed the Random Forest algorithm [46]. It is widely embraced in ensemble learning. It functions by creating numerous decision trees during the training phase and then deriving the mode of classes for classification tasks or the average prediction for regression tasks from these individual trees.

In [17], Random Forest classifiers were utilized to forecast OSAS severity, specifically focusing on categorizing AHI < 15 vs. AHI ≥ 15 and AHI < 30 vs. AHI ≥ 30. The results demonstrated a sensitivity of 80% and specificity of 46.67% for distinguishing AHI < 15 vs. AHI ≥ 15, and a sensitivity of 57.14% and specificity of 93.75% for AHI < 30 vs. AHI ≥ 30.

Wang et al. in [23] achieved outstanding results in accurately identifying apnea, with a specificity of 91.8%, a sensitivity of 89.93%, and an accuracy of 93.88%. This algorithm was chosen as the central model for the automated apnea screening system due to its superior capability in mitigating overfitting issues while delivering high classification accuracy and rapid training speed.

The study detailed in [28] employed Random Forest as a key machine learning algorithm for classifying respiratory events. The Random Forest technique constructs decision tree models through bootstrap training on the same dataset. Classification outcomes are determined via a majority vote from the decision tree algorithms comprising the Random Forest ensemble. The Random Forest model achieved impressive performance metrics, including an overall accuracy of 83%, sensitivity of 99%, specificity of 79%, and an F1 score of 81%.

In [37], a Random Forest model was trained using features extracted from ECG signals to detect patterns indicative of sleep apnea. The results yielded an Accuracy Score of 66.569%, Sensitivity of 51.679%, Specificity of 85.377%, False Positive Rate of 14.622%, and an AUC of 68.205%.

To complete this modeling approach in [39], the ApneaDetector system assesses various classification algorithms to detect sleep apnea events, including Decision Tree (DT), Naive Bayes (NB), Support Vector Machine (SVM), Random Forest (RF), and Adaptive Boost (ABT). These algorithms are compared based on their F1-scores and execution times to identify the most effective classifier for the system. Employing a 10-fold cross-validation scheme on the training set aids in selecting the optimal classifier for ApneaDetector. Ultimately, Random Forest (RF) is chosen as the classifier for ApneaDetector due to its high F1-score and efficient processing of sensor data collected from the smartwatch. With a precision of 0.97, recall of 0.98, and an F1-score of 0.98, RF demonstrates superior performance in accurately identifying sleep apnea events.

In the study cited as [28], Linear Discriminant Analysis (LDA) is highlighted as a pivotal algorithm. LDA computes the mean and covariance matrix for each class within the training data. Leveraging this information, it determines an optimal decision boundary aimed at maximizing class separation while minimizing variance within each class.

Within the paper’s context, LDA emerged as a sophisticated machine learning technique for respiratory event detection. Notably, it achieved remarkable performance metrics: an overall accuracy of 81%, sensitivity of 88%, specificity of 79%, and an F1 score of 81%.

Quadratic Discriminant Analysis, introduced in [28], stands as a classical statistical technique applied in classification tasks. It bears resemblance to Linear Discriminant Analysis (LDA) but offers greater adaptability in delineating decision boundaries among classes by presuming unique covariance matrices for each class. In the study, QDA was among the machine learning algorithms deployed for classifying respiratory events. The method exhibited strong performance with QDA, boasting an overall accuracy of 80%, sensitivity 92%, specificity 50%, F1 score 70% and a Cohen’s kappa of 0.47 for minute-by-minute respiratory event detection.

In three separate studies, the Support Vector Machine (SVM) algorithm was introduced as a method to identify the optimal hyperplane for effectively distinguishing between different classes within the feature space. In [23], the research revealed that the SVM model achieved an accuracy rate of 88.28%, with a specificity of 91.69% and a sensitivity of 83.94% in evaluating sleep apnea. Despite demonstrating commendable performance, the random forest model employed in the same study surpassed the SVM in terms of accuracy, specificity, and sensitivity. In the case of [28] SVM was used as one of the machine learning algorithms for classification in the respiratory event detection task. The proposed method achieved high performance using SVM, with an overall accuracy of 83% and a Cohen’s kappa of 0.53 for the minute-by-minute respiratory event detection. Finally, [37] the Support Vector Machine (SVM) was instrumental in the study’s automated sleep apnea detection. With an accuracy score of 69.131%, sensitivity of 53.341%, and specificity of 80.026%, SVM effectively classified sleep apnea events from single-channel ECG signals. Trained on ECG data features, SVM aimed to discern sleep apnea patterns by optimizing class separation within the feature space. Its integration into the ensemble of classifiers significantly enhanced the system’s accuracy. The SVM model’s balanced performance metrics underscore its pivotal role in the study’s quest for automated sleep apnea detection using machine learning techniques.

Gaussian Naive Bayes is a probabilistic algorithm that leverages Bayes’ theorem, assuming feature independence. It’s a simple and efficient method widely employed in classification tasks, as outlined in [29]. In the study, GNB was deployed to analyze 3-ACC, SpO2, and heart rate data collected from wearable devices. The algorithm operates under the assumption of Gaussian distribution in the data and computes class probabilities based on input features.

To assess GNB’s accuracy, the study measured the variance and average of the training data. Despite achieving an accuracy of approximately 89% in distinguishing between respiration and apnea, GNB’s accuracy suffered due to significant variance stemming from the accelerometer’s data structure.

K-Nearest Neighbors (KNN) stands out as another notable machine learning algorithm [47]. KNN operates by classifying or predicting a data point based on the majority class or average of its nearest neighbors in the feature space. It’s a straightforward yet robust algorithm rooted in the principle of data point similarity and has been the focus of two studies. In [23], the KNN model demonstrated an accuracy of 85.06%, specificity of 86.11%, and sensitivity of 83.72% when processing photoplethysmography (PPG) signals. On the other hand, in [29], KNN exhibited promising outcomes, achieving a remarkable accuracy of 95% while adhering to acceptable timing constraints for diagnosing sleep apnea. The algorithm’s proficiency in measuring similarity between observed data and learning data significantly contributed to its accuracy in predicting levels of sleep apnea.

In addition, the XGBoost algorithm was introduced in [23] and is recognized for its proficiency in addressing both regression and classification tasks. The research revealed that the XGBoost model exhibited an accuracy rate of 82.05%, with a specificity of 84.91% and a sensitivity of 78.42% in evaluating sleep apnea.

For the case of [37] the authors delve into automated sleep apnea detection, assessing the efficacy of various machine learning models for classification tasks. Among these models are Decision Tree (DT), which constructs a tree-like structure to partition data based on features, and its extension, Adaptive Decision Tree (ADT), which combines predictions from multiple weak classifiers to improve performance. Additionally, they investigate Voting Classifier (VC), which aggregates predictions from various classifiers to determine the most probable class, and Stacked Classifier (SC), an ensemble learning model that combines multiple base models to enhance prediction accuracy. Their findings reveal that while Decision Tree achieves the highest accuracy score at 82.902%, Stacked Classifier exhibits the most promising performance with an accuracy score of 74.315%, underscoring the importance of ensemble techniques in automating sleep apnea detection from single-channel ECG signals.

#### Neural Networks

This section addresses RQ4, which examines various neural networks models and their corresponding achievements. Table 8 presents a comprehensive overview of the distribution of different methods and metrics utilized in the analysis.

**Table 8:** Distribution of neural networks methods and metrics

Neural networks models	Percentage	References	Metric	*N*
Deep Neural Networks	8%	[22, 34]	Accuracy	2
Convolutional Neural Networks	36%	[18–21, 24, 27, 32, 36, 38]	Accuracy, precision, recall, and F1 score	9
Artificial Neural Network	4%	[29]	Accuracy, precision, recall, and F1 score	1
Recurrent Neural Networks	20%	[13, 14, 16, 25, 31]	Accuracy, sensitivity, F1, specificity and ROC-AUC	5
Feedforward Neural Networks	12%	[15, 17, 28]	Precision, recall, F1-score, sensitivity, and accuracy	3
Transfer Learning	16%	[12, 26, 30, 35]	Accuracy, precision, recall, Cohen’s kappa and F1 score	4
Hierarchical Deep Clustering	4%	[33]	Accuracy	1

In [22] a Deep Neural Network (DNN) was developed to harness a sensor-fusion approach for processing surrogate sonographic signals captured by a chest-worn sensor. The network architecture was specifically tailored to tackle a three-fold classification task, which involved predicting the overall signal quality, three breathing-related patterns, and three sleep-related patterns. Evaluation metrics revealed the AI model’s effectiveness in identifying sleep-related breathing events. The network exhibited an overall accuracy of 0.96 in distinguishing normal signals from corrupted ones. Notably, breathing patterns were predicted with greater precision (0.93) compared to sleep patterns (0.76). The model demonstrated slightly lower accuracy in predicting irregular breathing (0.88) than apnea (0.97). Within the sleep pattern category, differentiation between snoring (0.73) and noise events (0.61) proved to be less effective. In [34], the study employed a deep convolutional neural network (DNN) to classify sleep stages and detect sleep apnea using data from wrist-worn consumer sleep technologies (CST). This DNN, incorporating accelerometer and photoplethysmography signals, was structured with two distinct streams for classification tasks. Training and testing were conducted on internal datasets, revealing improved performance with raw data inputs, especially in accurately identifying apnea events during REM sleep. Despite a slight decrease in performance with CST data, the model still demonstrated effectiveness in sleep apnea detection.

Convolutional Neural Networks (CNN) is a type of deep learning algorithm commonly used for image recognition and classification tasks [48]. CNNs are characterized by their ability to automatically learn hierarchical patterns and features directly from the raw pixel data of images. In [18], CNNs are utilized to analyze spectrogram images extracted from physiological signals, enabling automated classification of various sleep stages and detection of apnea events. These CNNs undergo training with labeled data, where spectrogram images are paired with corresponding sleep stages and apnea events. This process enables the models to discern relationships between signal patterns and specific sleep-related phenomena, achieving an accuracy of 88.52%. In [19], a one-dimensional convolutional neural network (1D-CNN) was employed to discern and classify distinct breathing episodes from raw data collected by wearable sensors. The model comprised 34 layers, incorporating convolutional operations, batch normalization utilizing a ReLu kernel function, and four residual connections, each featuring two convolutional layers. The final layer of the model was fully connected and included both SoftMax and moment components. Impressively, the model achieved significant performance metrics: an accuracy of 0.95, recall of 0.97, and F1 score of 0.93 for apnea, and an accuracy of 0.91, recall of 0.86, and F1 score of 0.87 for expiration, among other evaluation metrics. In [20], a CNN was employed to identify breath patterns across various body positions and conditions, demonstrating consistent performance unaffected by changes in position. The model exhibited a sensitivity of 97.6%, specificity of 79.7%, and maintained an average accuracy exceeding 96%. In [21], the study deployed deep learning models for both binary and multi-event classification, as well as for the detection of breathing patterns. For binary classification, a 1D-CNN akin to an AlexNet was utilized, comprising 6 convolutional layers and 4 fully connected layers. Regarding multi-event classification, a deep learning model was employed, incorporating residual blocks with a filter length of 384. Similarly, for the detection of breathing patterns, a 1D-CNN was utilized, employing residual blocks with a filter length of 384. Evaluation metrics for the multi-event classification model revealed F1 scores of 87% for CSA, 56% for normal breathing, 83% for coughing, 55% for OSA, 53% for sighing, and 55% for yawning on the testing dataset. Moreover, the detection model achieved notable F1 scores (86%, 95%, and 72%, respectively) for CSA, normal breathing, and coughing. In [24], they present the application of a 1-D convolutional neural network (1DCNN) in deep learning for analyzing respiratory signals. The AI model is used for disease classification with an accuracy of 96.67% and identity recognition with an accuracy of 93.67%. In [27] researchers investigated a fusion of convolutional and recurrent neural network layers to extract features from rPPG signals and identify respiratory events during sleep. The deep learning model attained ROC-AUC values of 0.84, 0.86, and 0.85 for mild, moderate, and severe OSA, respectively. In their work [32], the authors emphasize the effectiveness and promise of CNN-based methodologies in precisely distinguishing apnea from non-apnea episodes. Particularly noteworthy is the outstanding performance of the CNN model BBHBA3, which demonstrates remarkable metrics including a sensitivity of 89.2%, specificity of 92.8%, an Area Under the Curve (AUC) of 96.4%, and accuracy reaching 90.1%. These results underscore the significant role of AI-driven techniques in elevating diagnostic accuracy and enhancing patient care standards in the domain of sleep medicine. In [36], the study proposes the one-dimensional multiscale bidirectional temporal convolutional network (1D-MsBiTCNet) as an AI model for detecting sleep apnea using wearable photoplethysmography (PPG) data from commercial bracelets. The model, based entirely on convolutional neural networks, integrates multiscale feature extraction and temporal dependence representation techniques. To combat overfitting and class imbalance during training, it employs regularized dropout (RD) and logit adjustment (LA) methods. Demonstrating high accuracy, sensitivity, and specificity, the 1D-MsBiTCNet excels in per-segment and per-recording detection of sleep apnea and accurately quantifies the apnea-hypopnea index (AHI). For per-segment detection, it achieves 82.76% accuracy, 71.58% sensitivity, and 86.74% specificity, while for severe sleep apnea syndrome (SAS), it reaches 97.83% accuracy, 88.89% sensitivity, and 100.00% specificity. Moreover, it yields a mean absolute error of 5.44 in AHI prediction. Overall, the 1D-MsBiTCNet offers a promising approach for automatic sleep apnea detection, with potential for widespread application. In [38], the study introduces a pioneering approach to detect sleep apnea events utilizing a 1-dimensional convolutional neural network (1D-CNN) called SomnNET, which analyzes peripheral oxygen saturation (SpO2) signals collected from wearable devices. Operating at a high resolution on a per-second basis, SomnNET aims to enhance the accuracy of sleep apnea detection in comparison to existing methodologies. Notably, the SomnNET network achieved an impressive accuracy of 97.08% in identifying sleep apnea events, surpassing several state-of-the-art algorithms with lower resolutions. Furthermore, the research investigates the potential of model pruning and binarization to mitigate computational complexity. The pruned network, with 80% sparsity, exhibited an accuracy of 89.75%, while the binarized network achieved an accuracy of 68.22%.

Artificial Neural Networks (ANNs) represent computational models engineered to emulate the structure and functionality of biological neural networks, as outlined in [29]. They serve as pivotal components in various AI applications, facilitating tasks such as pattern recognition, classification, regression, and more. In the study, the ANN model’s architecture comprised multiple layers of neurons, featuring specific configurations of hidden layers and nodes. Notably, the hidden layer configuration encompassed three layers, each housing 600 nodes. The arrangement of these hidden layers significantly influences the performance of the ANN model. Although the overall accuracy of the ANN model hovered around 71% for each class, it notably achieved a higher accuracy rate of 92% for distinguishing between normal respiration and apnea. These performance metrics shed light on the model’s proficiency by accurately categorizing different classes of sleep apnea based on the bio-signals captured by the wearable device.

Recurrent Neural Networks are a class of artificial neural networks that are designed to handle sequential data by maintaining an internal memory state [49]. Unlike feedforward neural networks, where information flows only in one direction, RNNs have connections that loop back on themselves, allowing them to retain information about previous inputs in the sequence.

In [13], researchers utilized the Long Short-Term Memory (LSTM) model, a form of recurrent neural network (RNN) architecture well-suited for analyzing sequential data. The LSTM-RNN model was applied to classify and analyze physiological signals, specifically targeting the detection of sleep apnea-hypopnea syndrome (SAHS). Comprising three LSTM loop layers and four fully connected layers with nodes set at 128, 64, 32, and 1, the LSTM-RNN model showcased notable accuracy in identifying SAHS, achieving an average recognition rate of up to 84–85%. Furthermore, in [14], researchers employed the LSTM model in conjunction with the MobileNet V1 architecture and Gated Recurrent Unit (GRU). This amalgamation enabled the model to proficiently extract features from ECG signals and scrutinize the temporal connections within the data, facilitating the detection of obstructive sleep apnea. The model attained an accuracy of 90.29%, sensitivity of 90.01%, and specificity of 90.72%, underscoring its efficacy in identifying sleep apnea from ECG signals. In [16], researchers also employed an LSTM model trained on bio-impedance data gathered from the chest using a portable device to analyze various types of sleep apnea events. They achieved a sensitivity of 58.4%, specificity of 76.2%, and accuracy of 72.8%, respectively. In [25] They employed the Convolutional LSTM (ConvLSTM) network, which integrates convolutional neural network (CNN), Long Short-Term Memory (LSTM) layer, and fully connected layer components. The CNN layers were tasked with extracting features from the tracheal movement signals, while the LSTM layer encoded the temporal dependencies within the signals. Subsequently, the fully connected layer facilitated the classification of signals into respiratory event or non-event categories. The outcomes revealed an F1 score range of 12–71% for various groups of sleep apnea severity within the event-by-event detection algorithm. Furthermore, the sensitivity, specificity, and accuracy of diagnosing sleep apnea stood at 81%, 87%, and 84%, respectively. In [31], in contrast to the methodologies in the other four studies, researchers employed a Recurrent Convolutional Neural Network (RCNN) model. This model was trained on 145 input features extracted from physiological signals and actigraphy metrics. These features were predominantly derived from signal quality metrics during hand-annotated events to ensure precise classification of apnea and hypopnea events. Utilizing the soft sternal patch system, the model accurately detected apneas and hypopneas with 100% sensitivity and 95% precision.

Feedforward Neural Networks (FNNs) represent a fundamental category of artificial neural networks characterized by the absence of cyclic connections among neurons, thereby excluding feedback loops or recurrent connections [50]. In a specific study denoted by [15] the FNN model underwent training using various combinations of ECG sensor and pulse oximeter data. Notably, it exhibited superior accuracy compared to single-sensor models, achieving an overall accuracy surpassing 87%, a sensitivity of 88%, and an F1 score of 89%. The FNN architecture employed in the research comprises three hidden layers, incorporating two input variables and a single-unit output layer. Activation functions, namely ReLU in the hidden layers and sigmoid in the output layer, were leveraged for classification purposes. In [17], the utilization of Multilayer Perceptron (MLP), a subtype of Feedforward Neural Network (FNN), was highlighted. MLPs feature multiple layers of interconnected nodes, encompassing an input layer, one or more hidden layers, and an output layer, facilitating unidirectional information flow devoid of cyclic or recurrent connections. The MLP classifiers underwent training utilizing various descriptors, including Age, Gender, BMI, Sleep Efficiency (SE), Total Sleep Time (TST), Sleep Fragmentation Index (SFI), Wake After Sleep Onset (WASO), Number of Awakenings (Naw), mean Heart Rate during sleep (mHRs), and mean Heart Rate during sleep arousal (mHRw). Initialization of the MLP classifiers involved configuring specific parameters, such as the number of estimators and default settings sourced from the sci-kit-learn library. Additionally, a feature importance analysis was conducted to assess each descriptor’s contribution to the classification model.

The metrics outcomes for the MLP classifiers underscored their effectiveness in predicting various ranges of the Apnea-Hypopnea Index (AHI) thresholds. Sensitivity, indicative of the ability to accurately identify true positives, exhibited a range from 73.33 to 85.71% across AHI thresholds of < 5 vs. ≥ 5, <15 vs. ≥ 15, and < 30 vs. ≥ 30. Specificity, representing the capability to precisely identify true negatives, ranged from 41.67 to 66.67% for the same thresholds. Positive Predictive Value (PPV) spanned from 30.77 to 88.46%, while Negative Predictive Value (NPV) ranged from 46.15 to 94.87%. The Diagnostic Odds Ratio (DOR), measuring the ratio of true positive results to false positive results, varied from 1.96 to 8.22, indicating variability across thresholds. Moreover, the Matthews Correlation Coefficient (MCC), reflecting the correlation between observed and predicted classifications, ranged from 0.15 to 0.39, offering insights into the overall performance of the MLP classifiers across AHI thresholds. To complete this modeling approach, the study detailed in [28] also leveraged MLP models for respiratory event detection classification. Impressively, it achieved notable performance metrics, including an overall accuracy of 86%, sensitivity of 85%, specificity of 87%, and an F1 score of 83%.

Transfer Learning represents an artificial intelligence technique wherein knowledge acquired from training a model on a specific task is transferred and applied to a related task, thereby bypassing the need to initiate the learning process from scratch [51]. By harnessing the knowledge encapsulated in pre-trained models, transfer learning accelerates convergence and enhances performance. In [12], the utilization of the 1D-MMResSNet model implies the incorporation of a pre-trained ResNet model. The 1D-MMResSNet model, a deep neural network, integrates multitask learning, deep metric learning, and supervised representation learning methodologies. Its primary objective is to capture temporal dependencies within PPG signals, thereby enhancing the accuracy of sleep apnea detection. The study’s findings indicate that the proposed 1D-MMResSNet + CS model achieved notable performance metrics, including an accuracy of 81.32%, sensitivity of 70.27%, specificity of 85.81%, precision of 63.12%, F1 score of 66.50%, and an AUC of 0.8641. In a separate study, outlined in [26] the utilization of ResNet models was proposed. Here, three models—ResNet18, ResNet34, and ResNet50—were compared to evaluate ECG data for detecting abnormal breathing patterns and estimating the apnea-hypopnea index (AHI). ResNet18 exhibited superior performance among the models, boasting an average Cohen’s kappa coefficient of 0.57, a Pearson’s correlation coefficient of 0.87 between PSG and EAHI, and AUPR values ranging from 0.65 to 0.91 across different datasets. While explicit metrics for ResNet34 and ResNet50 were not provided, they were included in the comparative analysis to gauge the effectiveness of the screening method. Notably, ResNet18 excelled in identifying abnormal breathing events and classifying patients based on AHI values, underscoring its potential for enhancing the screening process for sleep disorders. Additionally, ResNet models were suggested as potent tools for improving the analysis of ECG data to identify sleep-related abnormalities. In [30], the AI model architecture integrates ResNeXt with squeeze-and-excitation network modules for detecting respiratory events. Furthermore, it combines a Transformer with CNN for the classification of sleep stages. The findings indicate moderate to substantial agreement between the Belun Ring’s derived apnea-hypopnea index (bAHI) and PSG-AHI, with accuracies ranging from 0.85 to 0.91. In [35], examines the application of deep transfer learning in automated single-lead EEG sleep staging despite discrepancies in channels and participant demographics. The research assesses diverse training methodologies (pre-training, training from scratch, and fine-tuning) across different participant groups (healthy individuals, those with OSA, insomnia, RBD) and EEG channels (F3-M2, F3-F4) to identify the optimal approach under conditions of data mismatches and constrained data availability.

The study employs the TinySleepNet model, optimized for automated sleep staging using single-channel EEG data. This computationally efficient variant of DeepSleepNet exhibits strong performance, with 1.3 million learned parameters capturing relevant patterns for sleep staging. The model’s architecture includes CNN and LSTM layers, achieving robust generalizability across diverse datasets and electrode derivations (κ values: 0.77–0.82). The research investigates training strategies—pre-training, training-from-scratch, and fine-tuning—to enhance TinySleepNet’s performance in automated sleep staging tasks.

#### Hierarchical Deep Clustering

Hierarchical Deep Clustering (HDC) models have emerged as a potent approach for unraveling intricate structures within large datasets. By amalgamating deep learning techniques with hierarchical clustering methods, HDC models offer a robust framework for data analysis. In [33], study introduces an AI model for sleep apnea detection, combining Hyperdimensional Computing (HDC) and 1D-BlockLBP encoding. Achieving 70.17% accuracy in detecting sleep apnea segments, the model demonstrates efficiency and effectiveness comparable to traditional methods. Notably, it features a significantly lower memory footprint, reduced latency, and energy savings on the ARM Cortex-M4 processor. Generalization testing on the PhysioNet-ECG dataset confirms superior performance over the baseline model, underscoring the scalability and potential of the HDC-based solution for long-term, home-based sleep apnea monitoring, thereby enhancing patient care feasibility.

## Conclusions

The review found that the main portable devices used are wristbands or sports watches, rings, chest bands, and patches. These allow the use of data such as photoplethysmography, electrocardiography, respiratory rhythm, oxygen saturation, body position and tracheal movements, rotational motion, mechanical vibrations, temperature, electromyography, electrooculography, and electroencephalography to develop models that enable the detection, categorization, and classification of different sleep apnea states. Despite the potential of these devices adhesive patches are the optimal choice for monitoring apnea syndrome, providing continuous data on vital signs like heart rate and oxygen saturation. While chest bands offer accurate heart rate monitoring, wrist-based bracelets are comfortable for daily health tracking. Compact rings are gaining popularity but may have limitations in measuring sleep stages accurately. The choice of wearable ultimately depends on individual preferences and the health metrics one aims to monitor.

Depending on the choice of device and its placement, both the type and quality of the data will vary, although the most used data include photoplethysmography, electrocardiogram, respiration rate, oxygen saturation, and body position with chest movements. The various types of data mentioned have shown that the most utilized data preprocessing stages include segmentation, feature extraction, and labeling, which are then used to train convolutional networks, recurrent networks, with LSTM being the most commonly used, and combine them with pre-trained transfer learning models.

In conclusion, the review underscores the significant advancements in wearable devices for monitoring sleep apnea, highlighting the diverse range of options available, including wristbands, adhesive patches, chest bands, and compact rings. Each device offers unique benefits and challenges, with adhesive patches emerging as the optimal choice for continuous vital sign monitoring. The utilization of convolutional and recurrent neural networks, augmented by pre-trained transfer learning models, demonstrates promising avenues for early sleep apnea diagnosis. Moreover, the importance of robust data preprocessing techniques cannot be overstated, as they play a pivotal role in training accurate and reliable models. By leveraging the advancements in wearable technology and deep learning methodologies, the field is poised to make significant strides in improving the detection and management of sleep apnea in clinical settings.

## Data Availability

No datasets were generated or analysed during the current study.

## References

[1] Abbasi A, Gupta SS, Sabharwal N, et al (2021) A comprehensive review of obstructive sleep apnea. Sleep Science 14:142. 10.5935/1984-0063.20200056 34381578 10.5935/1984-0063.20200056 PMC8340897

[2] Suzuki M (2022) Obstructive sleep apnea -consideration of its pathogenesis. Auris Nasus Larynx 49:313–321. 10.1016/J.ANL.2021.10.007 34763987 10.1016/j.anl.2021.10.007

[3] Jimborean G, Sarkozy HB, Szatmari M, et al (2023) Correlations between daytime sleepiness, arterial hypertension and the degree of apnea in patients with obstructive sleep apnea syndrome. Romanian Journal of Rhinology 13:182–187. 10.2478/RJR-2023-0026

[4] Schütz SG, Lisabeth LD, Hsu CW, et al (2021) Central sleep apnea is uncommon after stroke. Sleep Med 77:304–306. 10.1016/J.SLEEP.2020.08.025 32948418 10.1016/j.sleep.2020.08.025 PMC8492109

[5] Naughton M, Cistulli PA, De Chazal P, et al (2020) Big Data in sleep apnoea: Opportunities and challenges. Respirology 25:486–494. 10.1111/RESP.13669 31411796 10.1111/resp.13669

[6] Roland J, Espiritu D (2021) Health Consequences of Obstructive Sleep Apnea. Management of Obstructive Sleep Apnea 23–43. 10.1007/978-3-030-54146-0_3

[7] Shan S, Wang S, Yang X, et al (2022) Effect of adenotonsillectomy on the growth, development, and comprehensive cognitive abilities of children with obstructive sleep apnea: a prospective single-arm study. BMC Pediatr 22:1–7. 10.1186/S12887-022-03111-W/TABLES/4 35033050 10.1186/s12887-022-03111-w PMC8760659

[8] Caples SM, Anderson WMD, Calero K, et al (2021) Use of polysomnography and home sleep apnea tests for the longitudinal management of obstructive sleep apnea in adults: An American Academy of Sleep Medicine clinical guidance statement. Journal of Clinical Sleep Medicine 17:1287–1293. 10.5664/JCSM.9240/SUPPL_FILE/JCSM.9240.DS001.PDF 33704050 10.5664/jcsm.9240 PMC8314660

[9] Massie F, Van Pee B, Bergmann J (2022) Correlations between home sleep apnea tests and polysomnography outcomes do not fully reflect the diagnostic accuracy of these tests. Journal of Clinical Sleep Medicine 18:871–876. 10.5664/JCSM.9744 34710039 10.5664/jcsm.9744 PMC8883090

[10] Chiner E, Cánovas C, Molina V, et al (2020) Home Respiratory Polygraphy is Useful in the Diagnosis of Childhood Obstructive Sleep Apnea Syndrome. Journal of Clinical Medicine 2020, Vol 9, Page 2067 9:2067. 10.3390/JCM9072067 10.3390/jcm9072067 PMC7408887 32630238

[11] Lildal K, Kissow Lildal T, Boudewyns A, et al (2023) Aalborg Universitet Validity of in-lab and home respiratory polygraphy for detecting obstructive sleep apnea in children Validity of in-lab and home respiratory polygraphy for detecting obstructive sleep apnea in children. 10.1016/j.sleep.2023.01.016 10.1016/j.sleep.2023.01.016 36857990

[12] Shen Q, Yang X, Zou L, et al (2022) Multitask Residual Shrinkage Convolutional Neural Network for Sleep Apnea Detection Based on Wearable Bracelet Photoplethysmography. IEEE Internet Things J 9:25207–25222. 10.1109/JIOT.2022.3195777

[13] Ji X, Rao Z, Zhang W, et al (2022) Airline Point-of-Care System on Seat Belt for Hybrid Physiological Signal Monitoring. Micromachines (Basel) 13:. 10.3390/mi13111880 10.3390/mi13111880 PMC9694689 36363901

[14] Hemrajani P, Dhaka VS, Rani G, et al (2023) Efficient Deep Learning Based Hybrid Model to Detect Obstructive Sleep Apnea. Sensors 23:. 10.3390/s23104692 10.3390/s23104692 PMC10224467 37430605

[15] Hassan O, Paul T, Thakker R, et al (2022) A Multi-Sensor Based Automatic Sleep Apnea Detection System for Adults Using Neural Network Inference on FPGA. In: 2022 IEEE International Symposium on Medical Measurements and Applications, MeMeA 2022 - Conference Proceedings

[16] Van Steenkiste T, Groenendaal W, Dreesen P, et al (2020) Portable Detection of Apnea and Hypopnea Events Using Bio-Impedance of the Chest and Deep Learning. IEEE J Biomed Health Inform 24:2589–2598. 10.1109/JBHI.2020.2967872 31976919 10.1109/JBHI.2020.2967872

[17] Benedetti D, Olcese U, Bruno S, et al (2022) Obstructive Sleep Apnoea Syndrome Screening Through Wrist-Worn Smartbands: A Machine-Learning Approach. Nat Sci Sleep 14:941–956. 10.2147/NSS.S352335 35611177 10.2147/NSS.S352335 PMC9124490

[18] Kwon S, Kim HS, Kwon K, et al (2023) At-home wireless sleep monitoring patches for the clinical assessment of sleep quality and sleep apnea. Sci Adv 9:. 10.1126/sciadv.adg9671 10.1126/sciadv.adg9671 PMC10208583 37224243

[19] Rubavathy SJ, Suresh GR, Senthilkumar C, et al (2022) Wearable Sensor-Based Human Exhalation Rhythm Recognition using Deep Learning Neural network. In: Proceedings of the 2022 International Conference on Innovative Computing, Intelligent Communication and Smart Electrical Systems, ICSES 2022

[20] Tarim EA, Erimez B, Degirmenci M, Cumhur Tekin H (2023) A Wearable Device Integrated with Deep Learning-Based Algorithms for the Analysis of Breath Patterns. Advanced Intelligent Systems. 10.1002/aisy.202300174

[21] McClure K, Erdreich B, Bates JHT, et al (2020) Classification and Detection of Breathing Patterns with Wearable Sensors and Deep Learning. SENSORS 20:. 10.3390/s20226481 10.3390/s20226481 PMC7698281 33202857

[22] Rossi M, Sala D, Bovio D, et al (2023) SLEEP-SEE-THROUGH: Explainable Deep Learning for Sleep Event Detection and Quantification From Wearable Somnography. IEEE J Biomed Health Inform 27:3129–3140. 10.1109/JBHI.2023.3267087 37058373 10.1109/JBHI.2023.3267087

[23] Wang SK, Xuan WP, Chen D, et al (2023) Machine Learning Assisted Wearable Wireless Device for Sleep Apnea Syndrome Diagnosis. BIOSENSORS-BASEL 13:. 10.3390/bios13040483 10.3390/bios13040483 PMC10136920 37185558

[24] Zhang H, Fu B, Su K, Yang Z (2023) Long-Term Sleep Respiratory Monitoring by Dual-Channel Flexible Wearable System and Deep Learning-Aided Analysis. IEEE Trans Instrum Meas 72:. 10.1109/TIM.2023.3289535

[25] Hafezi M, Montazeri N, Saha S, et al (2020) Sleep Apnea Severity Estimation from Tracheal Movements Using a Deep Learning Model. IEEE Access 8:22641–22649. 10.1109/ACCESS.2020.2969227

[26] Yeo M, Byun H, Lee J, et al (2022) Robust Method for Screening Sleep Apnea with Single-Lead ECG Using Deep Residual Network: Evaluation with Open Database and Patch-Type Wearable Device Data. IEEE J Biomed Health Inform 26:5428–5438. 10.1109/JBHI.2022.3203560 36048977 10.1109/JBHI.2022.3203560

[27] Papini GB, Fonseca P, van Gilst MM, et al (2020) Wearable monitoring of sleep-disordered breathing: estimation of the apnea–hypopnea index using wrist-worn reflective photoplethysmography. Sci Rep 10:. 10.1038/s41598-020-69935-7 10.1038/s41598-020-69935-7 PMC7421543 32782313

[28] Yeo M, Byun H, Lee J, et al (2022) Respiratory Event Detection during Sleep Using Electrocardiogram and Respiratory Related Signals: Using Polysomnogram and Patch-Type Wearable Device Data. IEEE J Biomed Health Inform 26:550–560. 10.1109/JBHI.2021.3098312 34288880 10.1109/JBHI.2021.3098312

[29] Jeon Y, Heo K, Kang SJ (2020) Real-Time Sleep Apnea Diagnosis Method Using Wearable Device without External Sensors. In: 2020 IEEE International Conference on Pervasive Computing and Communications Workshops, PerCom Workshops 2020

[30] Strumpf Z, Gu W, Tsai C-W, et al (2023) Belun Ring (Belun Sleep System BLS-100): Deep learning-facilitated wearable enables obstructive sleep apnea detection, apnea severity categorization, and sleep stage classification in patients suspected of obstructive sleep apnea. Sleep Health 9:430–440. 10.1016/j.sleh.2023.05.001 37380590 10.1016/j.sleh.2023.05.001

[31] Zavanelli N, Kim H, Kim J, et al (2021) At-home wireless monitoring of acute hemodynamic disturbances to detect sleep apnea and sleep stages via a soft sternal patch. Sci Adv 7:. 10.1126/sciadv.abl4146 10.1126/sciadv.abl4146 PMC8694628 34936438

[32] Abasi AK, Aloqaily M, Guizani M (2024) Bare-bones based honey badger algorithm of CNN for Sleep Apnea detection. Cluster Computing 2024 1–21. 10.1007/S10586-024-04309-6

[33] Chen T, Zhang J, Xu Z, et al (2024) Energy-Efficient Sleep Apnea Detection Using a Hyperdimensional Computing Framework Based on Wearable Bracelet Photoplethysmography. IEEE Trans Biomed Eng. 10.1109/TBME.2024.3377270 38483799 10.1109/TBME.2024.3377270

[34] Olsen M, Zeitzer JM, Richardson RN, et al (2024) A deep transfer learning approach for sleep stage classification and sleep apnea detection using wrist-worn consumer sleep technologies. IEEE Trans Biomed Eng 1–12. 10.1109/TBME.2024.3378480 10.1109/TBME.2024.3378480 38498753

[35] Van Der Aar JF, Van Den Ende DA, Fonseca P, et al (2023) Deep transfer learning for automated single-lead EEG sleep staging with channel and population mismatches. Front Physiol 14:1287342. 10.3389/fphys.2023.1287342 38250654 10.3389/fphys.2023.1287342 PMC10796543

[36] Zou L, Liu G (2024) Multiscale Bidirectional Temporal Convolutional Network for Sleep Apnea Detection Based on Wearable Photoplethysmography Bracelet. IEEE J Biomed Health Inform 28:1331–1340. 10.1109/JBHI.2023.3335658 37991905 10.1109/JBHI.2023.3335658

[37] Parbat D, Chakraborty M (2024) Multiscale entropy analysis of single lead ECG and ECG derived respiration for AI based prediction of sleep apnea events. Biomed Signal Process Control 87:105444. 10.1016/J.BSPC.2023.105444

[38] John A, Nundy KK, Cardiff B, John D (2021) SomnNET: An SpO2 Based Deep Learning Network for Sleep Apnea Detection in Smartwatches. Proceedings of the Annual International Conference of the IEEE Engineering in Medicine and Biology Society, EMBS 2021-January:1961–1964. 10.1109/EMBC46164.2021.9631037 10.1109/EMBC46164.2021.9631037 34891671

[39] Chen X, Fernandez-Mendoza J, Xiao Y, et al (2021) ApneaDetector: Detecting Sleep Apnea with Smartwatches. Proc ACM Interact Mob Wearable Ubiquitous Technol 5:. 10.1145/3463514

[40] Belun Technology - Shed Light on Your Sleep. https://beluntech.com/. Accessed 13 Dec 2023

[41] Lamberti JP (2020) Respiratory Monitoring in General Care Units. Respir Care 65:870–881. 10.4187/RESPCARE.07405 32457176 10.4187/respcare.07405

[42] Hafiz AM, Bhat GM (2020) A survey on instance segmentation: state of the art. Int J Multimed Inf Retr 9:171–189. 10.1007/S13735-020-00195-X/TABLES/3

[43] Singh D, Singh B (2020) Investigating the impact of data normalization on classification performance. Appl Soft Comput 97:105524. 10.1016/J.ASOC.2019.105524

[44] Elgendi M, Norton I, Brearley M, et al (2013) Systolic peak detection in acceleration photoplethysmograms measured from emergency responders in tropical conditions. PLoS One 8:. 10.1371/JOURNAL.PONE.0076585 10.1371/journal.pone.0076585 PMC3805543 24167546

[45] Yang X, Hu X, Zhou S, et al (2024) Interpolation-Based Contrastive Learning for Few-Label Semi-Supervised Learning. IEEE Trans Neural Netw Learn Syst 35:2054–2065. 10.1109/TNNLS.2022.3186512 35797319 10.1109/TNNLS.2022.3186512

[46] Genuer R, Poggi J-M (2020) Random Forests. 33–55. 10.1007/978-3-030-56485-8_3

[47] Cunningham P, Delany SJ (2021) k-Nearest Neighbour Classifiers - A Tutorial. ACM Computing Surveys (CSUR) 54:. 10.1145/3459665

[48] Ketkar N, Moolayil J (2021) Convolutional Neural Networks. Deep Learning with Python 197–242. 10.1007/978-1-4842-5364-9_6

[49] Sherstinsky A (2020) Fundamentals of Recurrent Neural Network (RNN) and Long Short-Term Memory (LSTM) network. Physica D 404:132306. 10.1016/J.PHYSD.2019.132306

[50] Hemeida AM, Hassan SA, Mohamed AAA, et al (2020) Nature-inspired algorithms for feed-forward neural network classifiers: A survey of one decade of research. Ain Shams Engineering Journal 11:659–675. 10.1016/J.ASEJ.2020.01.007

[51] Zhuang F, Qi Z, Duan K, et al (2021) A Comprehensive Survey on Transfer Learning. Proceedings of the IEEE 109:43–76. 10.1109/JPROC.2020.3004555

